# Metabolomic study of saxitoxin analogues and biosynthetic intermediates in dinoflagellates using ^15^N-labelled sodium nitrate as a nitrogen source

**DOI:** 10.1038/s41598-019-39708-y

**Published:** 2019-03-05

**Authors:** Yuko Cho, Shigeki Tsuchiya, Takuo Omura, Kazuhiko Koike, Hiroshi Oikawa, Keiichi Konoki, Yasukatsu Oshima, Mari Yotsu-Yamashita

**Affiliations:** 1 Graduate School of Agricultural Science, Tohoku University, 468-1 Aramaki Aza Aoba, Aoba-ku, Sendai, Miyagi 980-8572 Japan; 2 Laboratory of Aquatic Science Consultant Co., LTD., 2-30-17, Higashikamata, Ota-ku, Tokyo 144-0031 Japan; 3 Graduate School of Biosphere Science, Hiroshima University, 1-4-4 Kagamiyama, Higashi-Hiroshima, 739-8528 Japan; 4 National Research Institute of Fisheries Science, Japan Fisheries Research and Education Agency, 2-12-4 Fukuura, Kanazawa, Yokohama, Kanagawa 236-8648 Japan; 5 Graduate School of Life Sciences, Tohoku University, 2-1-1 Katahira, Aoba-ku, Sendai 980-8577 Japan

## Abstract

A stable-isotope-labelling method using ^15^N-labelled sodium nitrate as a nitrogen source was developed for the toxic dinoflagellate *Alexandrium catenella*. The labelled saxitoxin analogues (STXs), their precursor, and the biosynthetic intermediates were analyzed by column-switching high-resolution hydrophilic interaction liquid chromatography with mass spectrometry. The low contents on Day 0, high ^15^N incorporation % of Int-C’2 and Int-E’ suggested that their turn-over rates are high and that the sizes of the pool of these compounds are smaller than those of the other intermediates. The experimentally determined isotopomer distributions showed that arginine, Int-C’2, 11-hydroxy-Int-C’2, Int-E’, GTX5, GTX4, C1, and C2, each existed as a combination of three populations that consisted of the non-labelled molecules and the labelled isotopomers representing molecules newly synthesized by incorporation of ^15^N assimilated from the medium with two different incorporation rates. The order of ^15^N incorporation % values of the labelled populations predicted by this model largely agreed with the proposed biosynthetic route. The stable-isotope-labelling method will be useful for understanding the complex mechanism of nitrogen flux in STX-producing dinoflagellates.

## Introduction

Paralytic shellfish poisoning is caused by consumption of shellfish contaminated with toxic marine dinoflagellates. The causative toxins are saxitoxin (STX) and its analogues, which are present in the organisms as a mixture of compounds that possess the same STX skeleton with different functionalities and stereochemistry^1,2^. STX analogues (STXs) act as potent neurotoxins that inhibit voltage-gated sodium channels^3^ and cause paralytic symptoms shortly after consumption. The causative organisms include species of dinoflagellates of the genus *Alexandrium*, *Gymnodinium catenatum*, and *Pyrodinium bahamense*^4–7^. The geographic occurrence area of STX-producing species continues to expand widely; the increase of algal bio-toxin problems in poorly monitored areas is worrying^8^. As the differences in inhibitory activity against sodium channels relate to differences in the chemical structure of each compound^9,10^, strains of the same species can show differing toxicities depending on the distinct molecular profiles of STXs that are synthesized^11^. Early warnings on toxicity would require knowing the biochemical processes responsible for the synthesis of STX-related compounds in dinoflagellates in response to environmental changes. However, the biosynthesis and metabolism of toxins in dinoflagellates remains poorly characterized.

**Figure 1:**
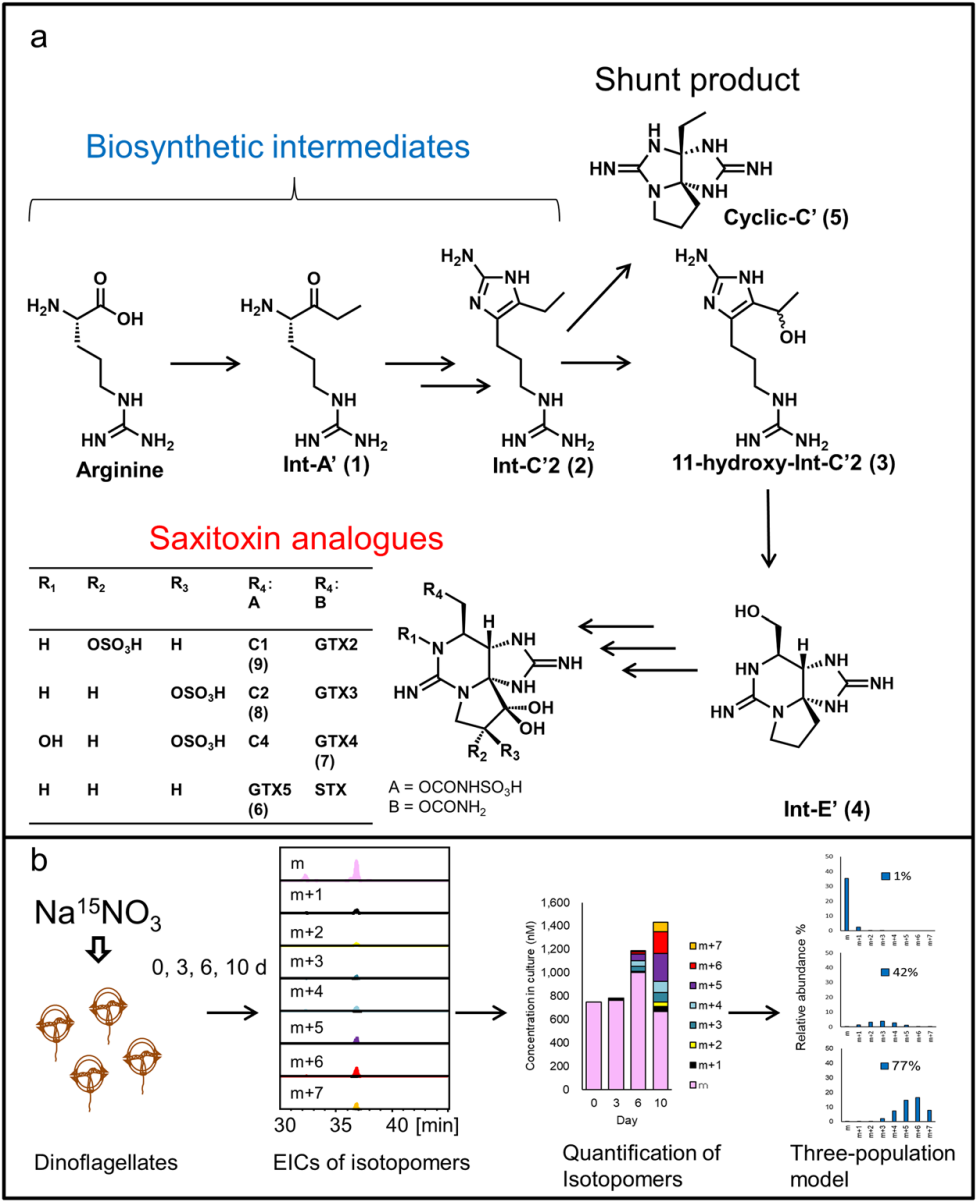
The proposed biosynthetic pathway of saxitoxin and its analogues (**a**) and the experimental workflow (**b**).

Cyanobacteria also produce STXs; after the cyanobacterial gene cluster for STX biosynthesis was identified by Kellmann *et al*., the biosynthetic intermediates were proposed based on the functions inferred from the gene sequences^12–14^. These substances were synthesized chemically and the labelled version of these substances evaluated in our laboratory for the incorporation of the label into cyanobacteria^15–18^. These studies revealed that Int-A’ (**1**), Int-C’2 (**2**), 11-hydroxy-Int-C’2 (**3**), and Int-E’ (**4**) are the genuine biosynthetic intermediates for STXs; on the other hand, Cyclic-C’ (**5**), a tri-cyclic guanidine compound generated from Int-C’2 (**2**), is a shunt product (Fig. 1). The column-switching high-resolution hydrophilic interaction liquid chromatography with mass spectrometry (HILIC-MS) method was developed to facilitate the highly specific and reliable identification and precise quantification of STX-related compounds^19,20^. The same biosynthetic intermediates identified in cyanobacteria were found in toxic sub-clones of *A. tamarense* and *A. catenella*, and these intermediates were not detected in a non-toxic sub-clone of *A. tamarense*^15,20^. While further molecular biological studies are needed, the unique characteristics of dinoflagellates present challenges for this research^21–23^.

As an alternative chemical approach, we exposed the STX-producing dinoflagellate *A. tamarense* to metabolic inhibitors (colchicine and 5-fluoro-2′-deoxy-uridine (FUdR)). Previous work showed that these inhibitors impaired toxin production by *A. tamarense*^24,25^. Subsequently, proteomic analysis of STX-producing and -non-producing *A. catenella* incubated with and without colchicine was reported^26^. Interestingly, none of the seven identified toxin biosynthetic enzymes showed differential expression in that analysis, implying that STX production might be regulated post-translationally by colchicine^26^. To characterize the mechanism of action of these metabolic inhibitors, we sought to define the biosynthetic step(s) that were being affected. However, a preliminary study quantifying the dinoflagellates’ STX biosynthetic intermediates in the presence and absence of the metabolic inhibitors did not yield clear results (data not shown). This lack of clarity may have reflected difficulty in distinguishing newly synthesized molecules from those already generated (prior to inhibition). Therefore, we initiated a metabolomic study of the toxin-related compounds.

Previously, *A. tamarense* feeding experiments performed by Shimizu *et al*.^27,28^ using organic compounds like [1-^13^C]-arginine and [1-^13^C]-histidine suggested that these molecules could not reach the compartmentalized reaction site(s) in dinoflagellates in an intact form. These results stimulated us to instead use labelled inorganic nitrogen for the dinoflagellate labelling experiment. In this context, high-resolution (HR) -LC-MS recently was shown to be useful for studies employing stable isotope labelling^29–31^.

Thus, the main purpose of this study was to develop a method for labelling STXs and their biosynthetic intermediates for metabolomic analysis using ^15^N-sodium nitrate medium for the culturing of the toxic dinoflagellate *A. catenella*. The analytical method was validated using a sample containing the completely labelled compounds and the non-labelled standards and then applied to the time-course study. The production of compounds’ isotopomers and their level were systematically examined over 10 days and new hypotheses about the dynamics of STX biosynthesis and metabolism were proposed based on these observation.

## Results and Discussion

The isotope patterns and isotopomer abundances were analyzed for the precursor (arginine), the biosynthetic intermediates from the early stages of the pathway (Int-A’ (**1**), Int-C’2 (**2**), 11-hyroxy-Int-C’2 (**3**) and Int-E’ (**4**)), the shunt product, Cyclic-C’ (**5**) and the main STX analogues (GTX5 (**6**), GTX4 (**7**), C2 (**8**) and C1 (**9**)) detected in the toxic *A. catenella* strain (120518KureAC)^20^ used in this study. The chemical structures and the putative biosynthetic pathway in dinoflagellates are shown in Fig. 1 and their isotope patterns following 10 days of culturing on ^15^N-sodium nitrate medium are shown in Supplementary Information: Figs S-1–S-3. All possible isotopomers and the experimentally obtained precise mass of each isotopomer showed good agreement with the respective theoretical value (Supplementary Information: Table S-1), except for m + 3 peak of Cyclic-C’ (**5**) which was affected by the interference. The MS/MS analyses were conducted using the signals corresponding to the completely labelled isotopomer as the parent ion. The MS/MS spectra and annotation for the compounds are shown in Supplementary Information: Figs S-4–S-12. The precise mass of fragment ions for labelled samples increased according to the number of ^15^N atoms incorporated, compared to the corresponding fragment ions for non-labelled sample. For example, the precise mass of the fragment ions for ^15^N_7_-labelled C2 (**8**) (*m/z* 385.0635: [M–SO_3_–H_2_O + H]^+^, 323.1222: [M–2SO_3_ + H]^+^, and 305.1092: [M–2SO_3_–H_2_O + H]^+^) agreed with the theoretical values (Δ 1.6 mDa: *m/z* 385.0619 calculated for C_10_H_16_^15^N_7_O_7_S^+^, Δ 6.6 mDa: *m/z* 323.1156 calculated for C_10_H_18_^15^N_7_O_5_^+^, and Δ 4.1 mDa: *m/z* 305.1051 calculated for C_10_H_16_^15^N_7_O_4_^+^). Thus, these signals were confirmed to be those of the completely labelled isotopomers.

For the validation of the analytical method, the culture after two months maintenance with ^15^N-labelled sodium nitrate as a nitrogen source was used to mix with the non-labelled standard solution. EICs were generated for all relevant isotopomers and the peak areas were calculated. The ^15^N incorporation percentages of the sample before adding the standard were 94.9 ± 0.3%, 98.6 ± 0.4%, 97.9 ± 1.6%, 94.7 ± 0.2%, 97.0 ± 0.9%, 95.4 ± 1.1%, and 95.6 ± 3.6% (mean ± standard deviation (SD), n = 3) for arginine, Int-A’ (**1**), Int-C’2 (**2**), GTX5 (**6**), GTX4 (**7**), C2 (**8**), and C1 (**9**), respectively (Supplementary Information: Fig. S-13). No effect of ^15^N incorporation on retention time was observed. The within-day repeatability of the retention time was high, and the difference in retention times between the standard and the mixed samples was 0–0.1 min. The recovery rates of the main toxins and the biosynthetic intermediates from 50 mg of Chromabond^R^ HILIC sorbent were determined using a standard mixture prepared at a concentration range like that observed experimentally in the cell extracts of dinoflagellate cultures. (See Supplementary Information and Table S-2 for the optimization of sample clean-up). The recovery rates of the standard of the mixed samples from 50 mg of Chromabond^R^ HILIC sorbent were 24, 37, 17, 27, 62, 18, and 54% for arginine, Int-A’ (**1**), Int-C’2 (**2**), C1 (**9**), C2 (**8**), GTX4 (**7**), and GTX5 (**6**), respectively. The matrix compounds in the Chromabond^R^ HILIC-SPE eluate suppressed the peak areas of most compounds that had been added in the eluate (93, 60, 88, 79, 80, and 81% for arginine, Int-A’ (**1**), C1 (**9**), C2 (**8**), GTX4 (**7**), and GTX5 (**6**), respectively), whereas enhancement (134%) was observed for Int-C’2 (**2**). The peak areas demonstrated relative standard deviations (RSDs) of 12, 3, 10, 12, 10, 4, and 13% for arginine, Int-A’ (**1**), Int-C’2 (**2**), C1 (**9**), C2 (**8**), GTX4 (**7**), and GTX5 (**6**), respectively. The RSDs of relative % of the areas (that is, the peak areas of the mono-isotopic ion expressed as a percentages of the total areas of all isotopomers) were less than or equal to 5% (3, 5, 2, 1, 5, and 3% for arginine, Int-A’ (**1**), Int-C’2 (**2**), C1 (**9**), C2 (**8**), and GTX5 (**6**), respectively), with the exception of GTX4 (**7**) (14%). The validated method was applied to study the time course of the incorporation of ^15^N into each STX-related compound.

In the time-course study, the substitution of nitrogen from ^14^N to ^15^N did not affect cell growth (Supplementary Information: Fig. S-14). Cells at the late stationary phase (known as the non-toxin-producing phase)^32^ were used as inocula. By this phase, dinoflagellate cultures are believed to have depleted the medium of nitrate^33^. In the first 3 days following the addition of fresh medium, cells were presumed to be in induction phase, given that no increase in cell density was observed through Day 3; the rest of the growth period (through Day 10) appeared to correspond to an exponential growth phase. By Day 10, the STXs in the cultures had accumulated to 2.5-fold higher levels (compared to Day 0) whether grown in ^14^N or ^15^N medium (Supplementary Information: Fig. S-15), suggesting that isotope substitution did not influence toxin production.

**Figure 2:**
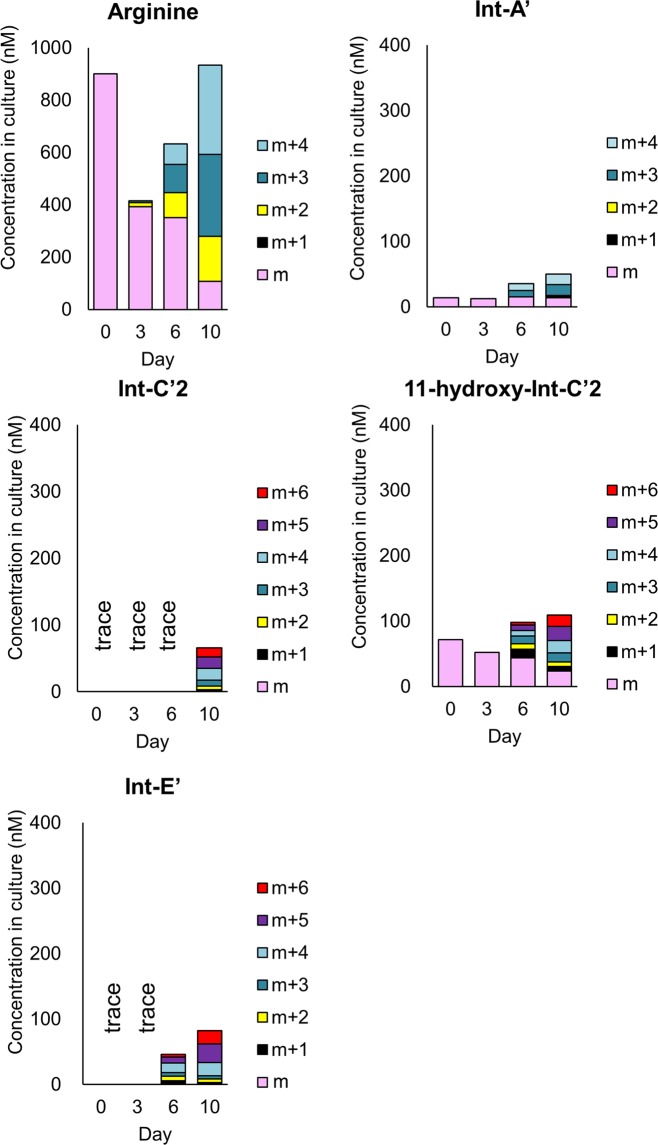
Quantification data of isotopomers of the biosynthetic intermediates and the shunt product in *A. catenella* cultured in ^15^N-NaNO_3_. Concentrations in culture (nM) for the each isotopomer of samples from 0, 3, 6, and 10 days (n = 3 for each day) after addition of ^15^N-labelled sodium nitrate-containing medium were calculated after removing the contribution of the naturally occurring stable isotope. The precursor: arginine, biosynthetic intermediates: Int-A’, Int-C’2, 11-hydroxy-Int-C’2, Int-E’ and the shunt product: Cyclic-C’.

**Figure 3:**
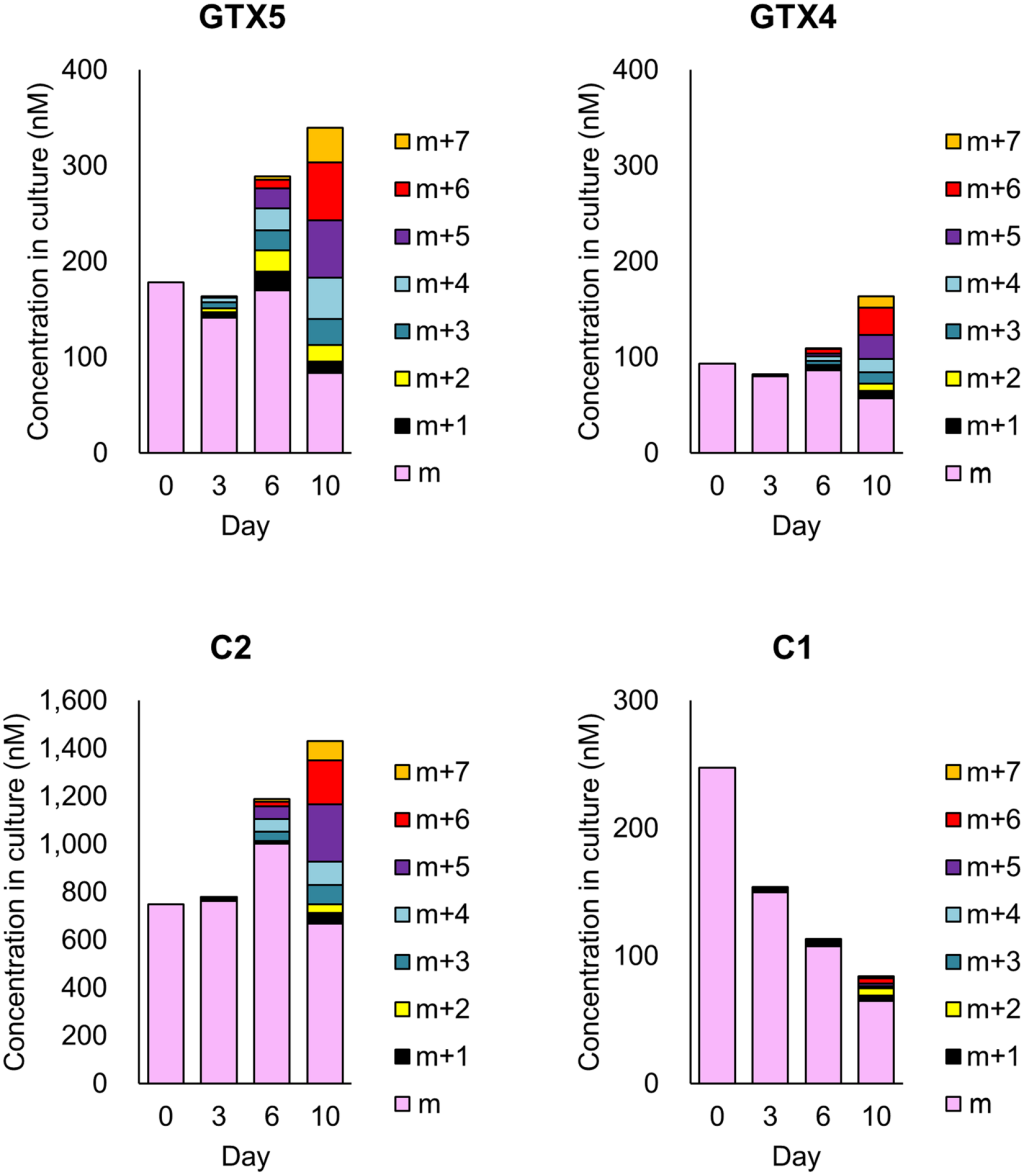
Quantification data of isotopomers of STXs in *A. catenella* cultured in ^15^N-NaNO_3_. Concentrations in culture (nM) for the each isotopomer of samples from 0, 3, 6, and 10 days (n = 3 for each day) after addition of ^15^N-labelled sodium nitrate-containing medium were calculated after removing the contribution of the naturally occurring stable isotope. STXs: GTX5, GTX4, C2 and C1.

The contents in culture for the each isotopomer of samples from 0, 3, 6, and 10 days (n = 3 for each day) after addition of ^15^N-labelled sodium nitrate-containing medium were calculated after removing the contribution of the naturally occurring stable isotope (details are shown in Supplementary Information: Table S-3) and the results are shown in Fig. 2 (the precursor, biosynthetic intermediates and the shunt product) and Fig. 3 (STXs). Int-C’2 (**2**) was quantified only on Day 10, since the intensities of the Int-C’2 (**2**) isotopomers on Days 0, 3 and 6 were too low for precise analysis. Int-E’ (**4**) was also quantified only Day 6 and Day 10. Cyclic-C’ (**5**) was not quantified, since the influence of the interference was observed. On Day 0, the precursor (arginine), the other intermediates (Int-A’ (**1**) and 11-hydroxy-Int-C’2 (**3**)) and STX analogues (GTX5 (**6**), GTX4 (**7**), C2 (**8**) and C1 (**9**)) were observed at the concentration of 901, 14, 72, 178, 93, 748 and 248 nM, respectively. The contents of the isotopomers of the biosynthetic intermediates and STXs on Day 3 did not differ significantly from Day 0, while that of arginine decreased to 392 and that of C1 (**9**) decreased to 150 nM. On Day 6 and Day 10, ^15^N incorporation was clearly observed for the precursor (arginine), the biosynthetic intermediates, STX analogues and the shunt product, and the total contents of all isotopomers increased, except for C1. The fact that the concentration of C1 decreased over time and the incorporation of ^15^N in C1 was very low suggested that the conversion of C1 to the downstream compound had proceeded more rapidly than the formation of C1 from the upper stream. Since C1 is believed to be formed from C2, its 11-β-isomer, through keto-enol equilibrium, the tautomerization rate may be very low in this period. The decrease of the contents of α-isomer at the position of C11 like C1 and GTX1 after passage had also been observed in *A. tamarense* previously^24^. Thus, it appeared that STX biosynthesis was induced between Day 3 and Day 6. The decrease of the contents for the non-labelled molecular species were observed for all compounds, except for GTX5 (**6**), GTX4 (**7**) and C2 (**8**) which showed the increase at Day 6. It suggested that these were produced by the non-labelled intermediates in the period of Day 3 to Day 6. In this period, the highest production rate was observed for C2 (**8**) (The production rates are shown in the Supplementary Information Table S-4).

The experimentally determined isotopomer distributions showed that these compounds each existed as a combination of at least two populations. The relative isotope abundance % values are shown in Supplementary Information: Figs S-16–17 (biosynthetic intermediates and the shunt product) and Figure S-18 (STXs). A similar pattern was reported for the peptides obtained by digestion of proteins in barley (*Hordeum vulgare*) plants grown in medium containing K^15^NO_3_ as a nitrogen source^29^. The population including the mono-isotopic ion is assumed to consist of the non-labelled molecules that might be a mixture of pre-existing and newly synthesized molecules generated from the non-labelled precursors. Previously, STXs were reported to be stored in the nucleus of the cell and periphery of small granules in *A. tamarense* (indicated as *Gonyaulax tamarensis*)^34^. *A. catenella*, the species used in the present study, is expected to store the STX analogues in such organelles. Moreover, amino acids in plant cells are known to be stored in the vacuole^35^. The level of non-labelled arginine observed in the present study suggested the presence of an arginine pool in *A. catenella*. The biosynthetic intermediate Int-A’ (**1**) and 11-hydroxy-Int-C’2 (**3**) also might be stored, since their isotopomer distribution showed a pattern like those of arginine and STXs. The low contents on Day 0, high ^15^N incorporation % and distinct isotopomer distribution of Int-C’2 (**2**) and Int-E’ (**4**) may reflect rapid turn-over of these compounds, resulting in depletion of the pool of non-labelled Int-C’2 (**2**) and Int-E’ (**4**). Int-C’2 (**2**) might be converted to the subsequent (downstream) biosynthetic intermediate via production of 11-hydroxy-Int-C’2 (**3**) or converted to the shunt product, Cyclic-C’ (**5**), immediately upon generation, preventing accumulation of Int-C’2 (**2**). Int-E’ (**4**) might also be converted to STX analogues via the subsequent (downstream) oxidized biosynthetic intermediates. A previous physiological study predicted that the size of the pool of amino acids and important metabolites might be related to the variability of toxin production under different nutrient conditions; biochemical reactions might be differentially affected by particular limiting nutrients or environmental factors in STX-producing dinoflagellates^36^. The present study experimentally demonstrated that the sizes of the pools of STX biosynthetic intermediates differ significantly. Further work will be needed to unveil the mechanism(s) whereby environmental factors regulate toxin synthesis.

**Figure 4:**
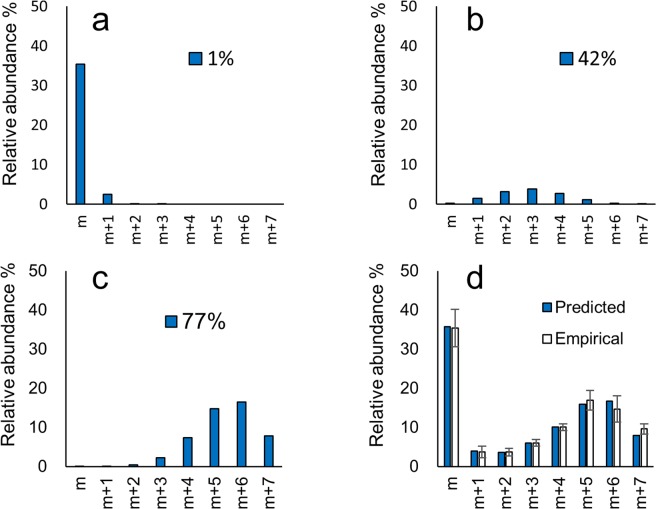
Predicted and empirical relative abundance % of each isotopomer of C2 [M-SO_3_ + H]^+^.

**Figure 5:**
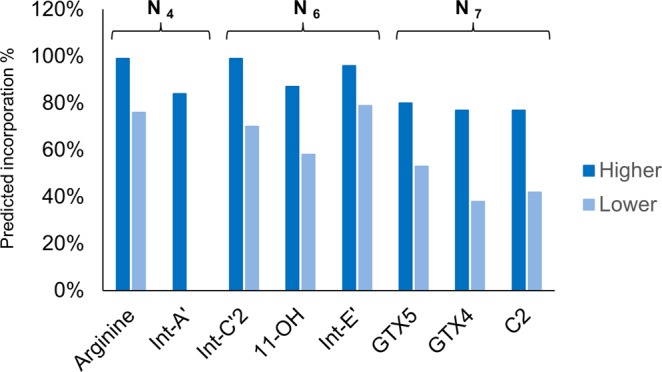
Incorporation % of the newly synthesized populations with higher and lower incorporation rates predicted by three-population model using the empirical values on Day 10.

**Figure 6:**
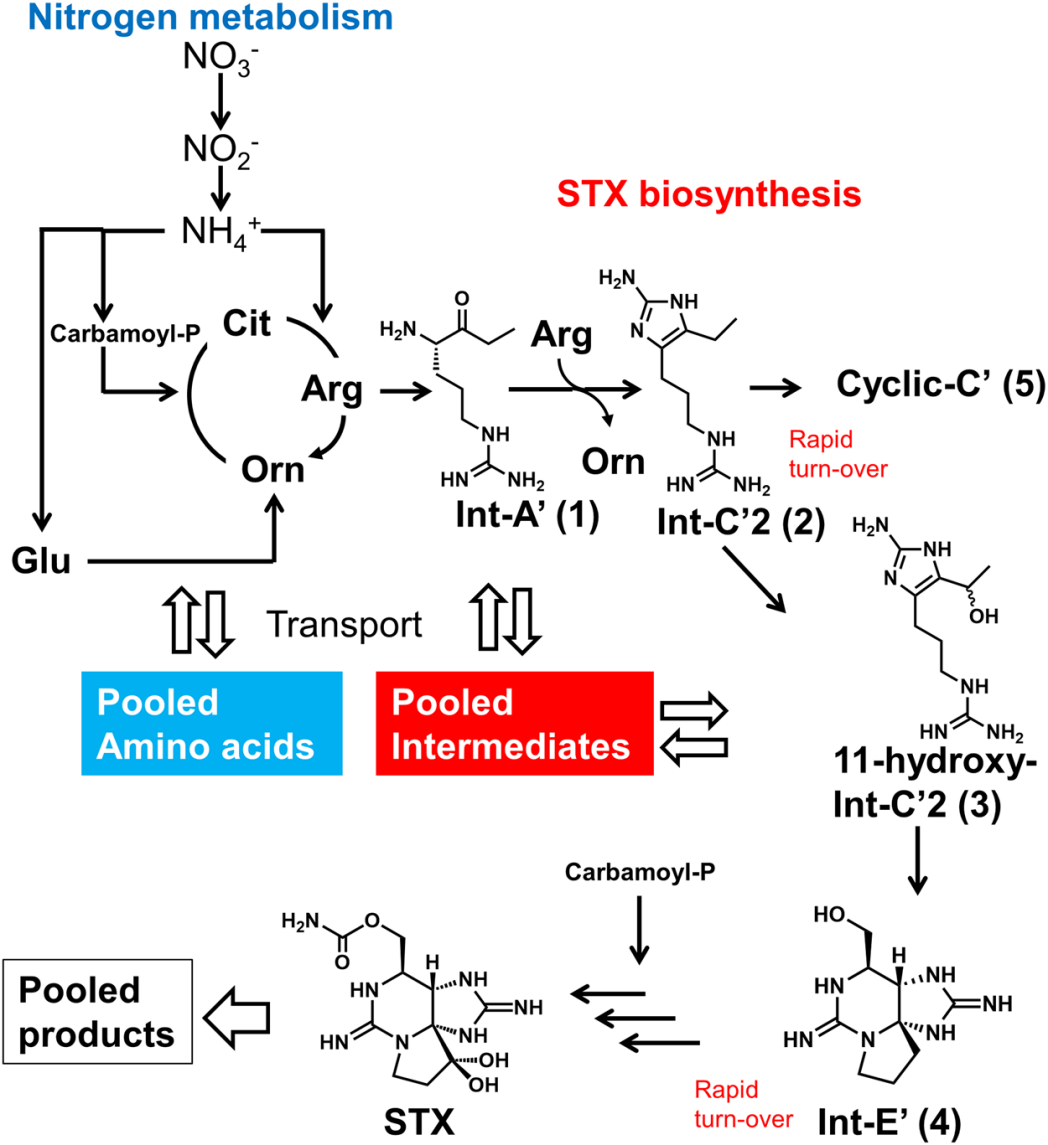
Possible mechanism of ^15^N incorporation into STX biosynthetic pathway.

Another population of molecules must include the labelled isotopomers representing molecules newly synthesized by incorporation of ^15^N assimilated from the medium. The relative isotopomer distribution of this population was predicted by hypothesizing that the probabilities of labelling of each nitrogen in the molecule are the same (stochastically predicted population). Since the preliminary results revealed that the two-population model and the empirical data showed a discrepancy, the existence of a third population was expected. The three-population model was successfully applied for all compounds except for Int-A’ (**1**) (Supplementary Information: Figs S-19–22 and Table S-5). As an example, the predicted and empirical relative abundance % of each isotopomer of C2 (**8**) [M–SO_3_ + H]^+^ on Day 10 is shown in Fig. 4. In this case, the populations with ^15^N incorporation at 1%, 42% and 77% were summed at the ratio of 38 : 13 : 49. The incorporation % of the newly synthesized populations with higher and lower incorporation rates predicted by three-population model using the empirical values on Day 10 is shown in Fig. 5. The order of ^15^N incorporation % values was largely consistent with the proposed biosynthetic route, the exceptions to this tendency were Int-C’2 (**2**) and Int-E’ (**4**). Int-A’ (**1**) is located one step beyond arginine, consistent with the proposed condensation of the new side chain onto arginine to afford Int-A’ (**1**)^37^. Int-A’ (**1**) is then postulated to be converted to Int-C’2 (**2**) by the transfer of an amidino functionality from arginine and subsequent cyclization. Int-C’2 (**2**) is hydroxylated to 11-hydroxy-Int-C’2 (**3**) and then cyclized to form Int-E’ (**4**). GTX5 (**6**) then would be produced from Int-E’ (**4**) by a process of four steps, including two hydroxylation reactions, a carbamoylation, and the transfer of a sulfate functionality. Alternatively, GTX4 (**7**) and C2 (**8**) are thought to be generated from Int-E’ (**4**) (via GTX3) by a six-step process. The existence of the lower- and higher-incorporation populations has been reported for galactolipids labelled by NaH^14^CO_3_ in the cyanobacteria *Anabaena variabilis* M3^38^. For instance, pooled non-labelled amino acids like glutamate may participate in STX biosynthesis in dinoflagellates (Fig. 6). Such non-labelled precursors might be used as substrates for biosynthetic enzymes after transport from a storage organelle to the reaction site where arginine and STX biosynthesis occur. Further studies of STX synthesis will be needed to test this hypothesis in dinoflagellates.

To consider the applicability of the labelling method, other dinoflagellates also were subjected to HILIC-MS analysis. The quantitative results for the toxic strain of *G. catenatum*, and those for the non-toxic strains of *A. insuetum* and *Prorocentrum triestinum*, are summarized in Table 1. For details please see Supplementary Information: Figure S-23–S-24 and Table S-6. The precursor, arginine, the biosynthetic intermediates, Int-A’ (**1**), Int-C’2 (**2**),11-hydroxy-Int-C’2 (**3**), Int-E’ (**4**) and the shunt product, Cyclic-C’ (**5**), along with STXs (C1 (**9**), C2 (**8**), C4, GTX5 (**6**), and GTX6), were detected in *G. catenatum*, whereas (aside from arginine) none of these molecules (or other STX analogues commonly produced by toxic dinoflagellates) were detected in *A. insuetum* and *P. triestinum*. The presence of the same biosynthetic intermediates in *G. catenatum* and in cyanobacteria, and the absence of these intermediates in the non-toxin producing species, strongly supports the hypothesis that the biosynthesis of STX analogues in the toxin-producing dinoflagellates and cyanobacteria proceeds via analogous pathways. We anticipate that the labelling method developed here will help to elucidate the mechanism(s) leading to the variations in toxin production observed under different environmental conditions.

**Table 1:** Intracellular contents (mean ± SD; n = 3) (fmol/cell) of the biosynthetic intermediates and STXs in the extracts of dinoflagellates.

	*A. insuetum*	*P. triestinum*	*G. catenatum*
	NIES-678	Ptri060930 Ohi	GC-18
C1	ND	ND	5.8 ± 0.9
C2	ND	ND	100 ± 3.9
C3	ND	ND	ND
C4	ND	ND	0.70 ± 0.10
GTX1	ND	ND	ND
GTX2	ND	ND	ND
GTX3	ND	ND	ND
GTX4	ND	ND	ND
GTX5	ND	ND	25 ± 1.7
GTX6	ND	ND	28 ± 0.9
dcGTX3	ND	ND	ND
neoSTX	ND	ND	ND
12β-deoxy-dcSTX	ND	ND	ND
Int-A'	ND	ND	0.27 ± 0.01
Int-C'2	ND	ND	0.87 ± 0.01
11-hydroxy-Int-C’2	ND	ND	trace
Int-E’	ND	ND	trace
Cyclic-C'	ND	ND	0.27 ± 0.01
Arginine	13.3 ± 2.6	2.0 ± 0.30	0.43 ± 0.03

The detection limits of the biosynthetic intermediate and STXs in *A. insuetum* and *P. triestinum* are shown in Supplementary Information: Table S-6.

In conclusion, the stable-isotope-labelling method newly developed in this study was successfully applied to the specific and precise analysis of labelled STX analogues and their biosynthetic intermediates in dinoflagellates. The contents of each isotopomer were determined and their time course change were analyzed. The low contents on Day 0, high ^15^N incorporation % and distinct isotopomer distribution of Int-C’2 (**2**) and Int-E’ (**4**) suggested that their turn-over rates are high and that the sizes of the pool of these compounds are smaller than those of the other intermediates. The three-population model was successfully applied, hypothesizing that the isotopomer distribution could be predicted by the combination of three binominal distributions at a different ratio. The order of predicted ^15^N incorporation % values was largely consistent with the proposed biosynthetic route, the exceptions to this tendency were Int-C’2 (**2**) and Int-E’ (**4**). The dinoflagellate used in this study, *A. catenella*, is one of the most-widely found STX-producing species in Japan. The technique described here also should be applicable to other STX-producing dinoflagellates. This method is expected to open the door for the study of the complex mechanisms whereby STX-producing dinoflagellates regulate toxin production and metabolism in response to environmental changes.

## Materials and Methods

### General information

Reagents and standards for primary metabolites used in the study were purchased from Sigma-Aldrich Co. (St. Louis, MO, USA) and Wako Pure Chemical Industries, Ltd. (Osaka, Japan). LC-MS-grade acetonitrile (Wako), ammonium formate, and formic acid (Optima™ LC/MS Grade, Fisher Scientific, Waltham, MA, USA) were used for LC-Q-Tof MS. Distilled water (MilliQ) purified with a Simplicity UV system (Millipore, Billerica, MA, USA) was used for all experiments. Acetic acid was super-special grade (Wako). LC-MS was performed using micrOTOF-Q II (ESI, Q-Tof) (Bruker Daltonics, Billerica, MA, USA) and API2000 (AB SCIEX, Foster City, CA, USA) instruments. HR MS was performed using the micrOTOF-Q II (ESI, Q-Tof).

### Dinoflagellate strains

*Alexandrium catenella* (120518KureAC) was originally isolated at Kure, Hiroshima, Japan, in 2012. *Alexandrium insuetum* strain NIES-678 was purchased from the National Institute for Environmental Studies Japan. *Prorocentrum triestinum* strain Ptri060930 Ohi was isolated at Ohi, Japan, in 2006. These strains were maintained and grown in modified T_1_ medium prepared in artificial seawater as 200-mL cultures in 250-mL plastic tissue culture flasks under the following culture conditions^39^: 12-h light/12-h dark photo-cycle with light provided by cool white bulbs (100–150 μmole photons m^−2^ s^−1^) at 15 °C. *Gymnodinium catenatum* strain GC-18 was isolated at Inokushi Bay in 2011 and maintained in f/2 medium^40,41^ prepared with aged sea water as 200-mL cultures in 500-mL glass culture flasks at 17 °C.

### Analytical standards for the biosynthetic intermediates and STXs

Standard solutions of STXs were prepared from natural sources in our laboratory, as described previously^20^. The synthetic standards for the biosynthetic intermediates were prepared in our laboratory^15–18^. L-Arginine was purchased from Nacalai Tesque Co. (Kyoto, Japan). Standards with a sulfate functionality at the C-11 position (i.e., GTX1 and GTX4 (**7**), GTX2 and GTX3, C1 (**9**) and C2 (**8**), C3 and C4, dcGTX2 and dcGTX3) were used as mixtures of stereoisomers at the equilibrium ratio. The 12-deoxy-dcSTXs were derivatized from dcSTX, and their concentrations were estimated using dcSTX as a standard. L-Arginine and STX analogues were dissolved in 0.05 M acetic acid. Int-A’ (**1**), Int-C’2 (**2**), 11-hydroxy-Int-C’2 (**3**), Int-E’ (**4**) and Cyclic-C’ (**5**) were dissolved in 0.5 M acetic acid. All standard stock solutions were kept at −30 °C.

### Sample preparation

The SPE treatment for sample preparation prior to the HR-HILIC-quadrupole time-of-flight (Q-Tof) MS was modified from the previously described method developed for STXs^42^. See Supplementary Information and Table S-2 for the optimization of sample clean-up. An aliquot (100 μL) of the extract after filtration was transferred to a new tube and mixed with three volumes of THF. The sample was loaded onto Chromabond^R^ HILIC (50 mg, MACHEREY-NAGEL) that had been hand-packed into a column and pre-conditioned with 200 μL of MilliQ water and 1 mL of THF. Following loading of the sample, the column was sequentially washed with 500 μL of THF, 500 μL of CH_3_CN, and 500 μL of CH_3_CN/water/HCOOH (95:5:0.1, v/v/v). The column was eluted with 200 μL of 0.2 M HCOOH and an aliquot of the eluate (10 or 20 μL) was subjected to LC-MS.

### The modified column-switching HR-HILIC-Q-Tof-MS and MS/MS method

The previously developed column-switching HR-HILIC-Q-Tof-MS method^19,20^, with slight modifications, was used for the simultaneous analysis of the labelled STX analogues and the labelled biosynthetic intermediates. Three guard column cartridges (Develosil C30-UG [4.0 × 10 mm, Nomura Chemical, Seto, Japan], TSKgel guardgel Amide-80 [5 µm, 2.0 × 10 mm, Tosoh, Tokyo, Japan], and SeQuant^®^ ZIC^®^-HILIC Guard [2.1 × 20 mm, Merck KGaA, Darmstadt, Germany]) were connected in tandem for the SPE process; this apparatus was used to connect the autosampler to the switching valve (EV700-100, Rheodyne) configured between the LC and the MS. A SeQuant^®^ ZIC^®^-HILIC column (PEEK, 2.1 × 150 mm, 5 µm) used for LC was used to connect the switching valve to the mass spectrometer. The position of the switching valve was set to ‘2’ (to waste) from 0.01 to 0.6 min, ‘1’ (to column) from 0.6 min to 50 min, and ‘2’ from 50 to 62 min. Mobile phase A was MilliQ H_2_O and mobile phase B was 200 mM HCOONH_4_ buffer containing 200 mM HCOOH/H_2_O/CH_3_CN (5:1.5:95, v/v/v). A gradient elution program was applied as follows: 0–3 min, 100% B; 3.1–6 min, 85% B; 6–10 min, 85–70% B; 10–12 min, 70% B; 12.1–15 min, 90% B; 15–34 min, 90–45% B; 34–39 min, 45% B; 39–40 min, 45–85% B; 40–49.9 min, 85% B; 49.9–50 min, 85–40% B; 50–51 min, 40% B; and 51.1–62 min, 100% B. The flow rate was set at 0.2 mL/min. The MS conditions were as follows: positive ionization mode; dry gas: nitrogen (7.0 L/min); drying temperature: 180 °C; nebulizer: 1.6 bar; capillary: 4,500 V. Extracted ion chromatograms (EICs) were obtained as the theoretical value ± 0.02 (Int-C’2 (**2**)) or ± 0.01 (all others). The theoretical values are summarized in Table S-1. High-resolution LC-MS/MS was performed in MRM mode setting, with [M + H]^+^ as the precursor ion. The precursor ion was *m/z* 179.1 (^15^N_4_-arginine), 191.1 (^15^N_4_-Int-A’), 217.1 (^15^N_6_-Int-C’2), 387.1 (^15^N_7_-GTX5), 403.1 (^15^N_7_-C2-SO_3_), and 419.1 (^15^N_7_-GTX4), with an isolation width of 2 Da. The sweeping collision energy was 40–120 eV for the biosynthetic intermediates and 19–56 eV for STX analogues.

### ^15^N incorporation study using ^15^N-labelled sodium nitrate as a nitrogen source

The toxic *A. catenella* strain (120518KureAC) was pre-cultured for 35 days in modified T_1_ medium containing ^14^N-sodium nitrate. An aliquot (125 mL) of this pre-culture was inoculated to an equal volume of modified T_1_ medium containing ^15^N-sodium nitrate as the sole nitrogen source (1 mM ^15^N-NaNO_3_, 0.1 mM NaH_2_PO_4_, 5 μM FeCl_3_·6H_2_O, 1 μM ZnSO_4_, 10 μM MnCl_2_, 0.5 μM Na_2_MoO_4_, 0.2 μM CoCl_2_, 0.01 μM CuSO_4_, and 2 nM H_2_SeO_3_, prepared in artificial seawater), yielding a 250-mL culture with an initial cell density of 7 × 10^3^ cells mL^−1^. An aliquot of the same pre-culture was harvested as the Day-0 sample. The remaining mixture was divided into nine separate cultures of 25 mL each in 50-mL tissue culture flasks. The cultures were incubated under the following culture conditions: 12-h light/12-h dark photo-cycle with light provided by cool white bulbs (100 μmole photons m^−2^ s^−1^) at 15 °C. Aliquots (20 mL for LC-MS study, and 0.1 mL for cell count) of each of 3 cell cultures were harvested on Days 3, 6, and 10 (i.e., in triplicate for a given day). As a control, the same procedure was performed using ^14^N-sodium nitrate as the nitrogen source.

### Calculation of peak areas removing the contribution of the naturally occurring stable isotope

The contribution of the naturally occurring stable isotopes in m + 1 and m + 2 were calculated using the theoretical natural abundance and the EIC peak area of m. The natural abundance % of each isotopomer was calculated by ChemDraw Professional, ver. 16 (PerkinElmer, Inc. Waltham, USA). The resulting values were subtracted from the experimentally obtained values for m + 1 and m + 2. The same procedure was repeated and each value after subtraction for each isotopomer was used for quantification. The details are shown in the Supplementary Information S35. An example of the calculation is shown in the Supplementary Information Table S-3.

### Prediction of isotope distributions by three-population model

The stochastically predicted isotope distributions were calculated using the published equation^43^ with modification. The probability of ^15^N incorporation was hypothesized to be same for all nitrogen atoms in the molecule. The peak area of each isotopomer without the naturally occurring stable isotope was calculated stochastically using the equation below.

$${{\rm{A}}}_{{\rm{M}}}={(1-{\rm{X}})}^{{\rm{n}}}$$

$${{\rm{A}}}_{{\rm{M}}+1}={\rm{X}}{}_{{\rm{x}}}\,(1-{\rm{X}}){}^{({\rm{n}}-1)}\,{\rm{x}}\,{}_{{\rm{n}}}{\rm{C}}_{1}$$

$${{\rm{A}}}_{{\rm{M}}+{\rm{k}}}(2\le {\rm{k}}\le {\rm{n}})={{\rm{X}}}^{{\rm{k}}}\,{\rm{x}}\,{(1-{\rm{X}})}^{({\rm{n}}-{\rm{k}})}{\rm{x}}\,{}_{{\rm{n}}}{\rm{C}}_{{\rm{k}}}$$

M = molecular number, A_M_ = peak area of the ion (*m/z* = M), X = average incorporation rate, n = maximum number of the labelled nitrogens, _x_C_y_ = combination that chooses y from x (x, y: integer).

The total peak area of each isotopomer was divided by the sum of the total peak area of all isotopomers to obtain the distribution %. The isotopomer distribution was hypothesized to be a combination of three binominal distributions with different ^15^N incorporation rates (p, q, r) at a ratio of A : B : C. The optimal ratio and ^15^N incorporation rates were calculated to minimize the sum of differences for each isotopomer abundance % from the corresponding empirical value by changing each of the input parameter in a systematic way (e.g. changing one parameter by 1% until the sum of differences is minimized, then changing another parameter by 1%). The calculation was performed using JavaScript with Eclipse.

## Supplementary information

Supplementary Information

## Data Availability

All data generated or analysed during this study are included in this published article (and its Supplementary Information file).
