# Supplementary material for: Metabolomic study of saxitoxin analogues and biosynthetic intermediates in dinoflagellates using 15N-labelled sodium nitrate as a nitrogen source
Source: Sci Rep. 2019 Mar 5;9:3460. doi: 10.1038/s41598-019-39708-y (PMC6401167; doi:10.1038/s41598-019-39708-y)
Supplement: Supplementary file 1 — Supplementary Information [file 41598_2019_39708_MOESM1_ESM.pdf]

Supplementary Information

Metabolomic study of saxitoxin analogues and biosynthetic intermediates in dinoflagellates using ^{15}N-labelled sodium nitrate as a nitrogen source

Yuko Cho*, [1], Shigeki Tsuchiya [1], Takuo Omura[2], Kazuhiko Koike[3], Hiroshi Oikawa[4], Keiichi Konoki[1], Yasukatsu Oshima[5, **] and Mari Yotsu-Yamashita[1]

[1] Graduate School of Agricultural Science, Tohoku University, 468-1 Aramaki Aza Aoba, Aoba-ku, Sendai, Miyagi, 980-8572, Japan
[2] Laboratory of Aquatic Science Consultant Co., LTD. 2-30-17, Higashikamata, Ota-ku, Tokyo, 144-0031, Japan
[3] Graduate School of Biosphere Science, Hiroshima University, 1-4-4 Kagamiyama, Higashi-Hiroshima, 739-8528, Japan
[4] National Research Institute of Fisheries Science, Japan Fisheries Research and Education Agency, 2-12-4 Fukuura, Kanazawa, Yokohama, Kanagawa 236-8648, Japan
[5] Graduate School of Life Sciences, Tohoku University, 2-1-1 Katahira, Aoba-ku, Sendai 980-8577, Japan.

Table of contents

Figure S-1. Isotope patterns of the labelled biosynthetic intermediates (arginine, Int-A', and Int-C'2) on Day 10 S3
Figure S-2. Isotope patterns of the labelled biosynthetic intermediates (11-hydroxy-Int-C'2, Int-E', and Cyclic-C') on Day 10 S4
Figure S-3. Isotope patterns of the labelled STX analogues (C1, C2, GTX4, and GTX5) on Day 10 S5
Figure S-4. MS/MS spectra of non-labelled (A) and perfectly labelled (B) arginine S6
Figure S-5. MS/MS spectra of non-labelled (A) and perfectly labelled (B) Int-A' S7
Figure S-6. MS/MS spectra of non-labelled (A) and perfectly labelled (B) Int-C'2 S8
Figure S-7. MS/MS spectra of non-labelled (A) and perfectly labelled (B) Int-C'2 S9
Figure S-8. MS/MS spectra of non-labelled (A) and perfectly labelled (B) Int-C'2 S10
Figure S-9. MS/MS spectra of non-labelled (A) and perfectly labelled (B) Int-C'2 S11
Figure S-10. MS/MS spectra of non-labelled (A) and perfectly labelled (B) GTX5 S12
Figure S-11. MS/MS spectra of non-labelled (A) and perfectly labelled (B) C2-SO_3 S13
Figure S-12. MS/MS spectra of non-labelled (A) and perfectly labelled (B) GTX4 S14
Figure S-13. Relative abundance % of each isotopomer of the biosynthetic intermediates in *A. catenella* after a 2-month passage in ^{15}N-$NaNO_3$ medium S15
Figure S-14. Growth curve of *A. catenella* cultured with ^{15}N-NO_3 or ^{14}N-NO_3 S16
Figure S-15. Day-10 toxin contents of *A. catenella* cultured in ^{15}N-$NaNO_3$ or ^{14}N-NO_3 S17
Figure S-16. Relative % of peak area of each isotopomer of the precursor and the biosynthetic intermediates at 3, 6, and 10 days after the addition of ^{15}N-NO_3 medium to *A. catenella* S18
Figure S-17. Relative % of peak area of each isotopomer the biosynthetic intermediates, 11-hydroxyl-Int-C'2 and Int-E' at 3, 6, and 10 days after the addition of ^{15}N-NO_3 medium to *A. catenella* S19
Figure S-18. Relative % of peak of each isotopomer of the STXs at 3, 6, and 10 days after the addition of ^{15}N-NO_3 medium in *A. catenella* S20
Figure S-19. Three populations stochastically predicted by empirical relative % of each isotopomer on Day 6 S21
Figure S-20. Three populations stochastically predicted by empirical relative % of each isotopomer on Day 10 S22
Figure S-21. Three populations stochastically predicted by empirical relative % of each isotopomer on Day 6 S23
Figure S-22. Three populations stochastically predicted by empirical relative % of each isotopomer on Day 10 S24
Figure S-23. HILIC-MS-MRM chromatogram of toxic *G. catenatum* extract S25
Figure S-24. HILIC-MS-MRM chromatogram of non-toxic *A. insuetum* extract S26
Table S-1. The theoretical values for the isotopomers with different number of ^{15}N S27
Table S-2. Clean up conditions employed for Chromabond^R HILIC and the resulting recovered compounds S28
Table S-3. Example of calculation of peak areas removing the contribution of the naturally occurring stable isotope S29
Table S-4. Production rate (nM/d) S30
Table S-5. ^{15}N incorporation and their ratio calculated by three-population model for the isotopomer distribution % of Day 6 and Day 10 S31
Table S-6. The detection limits (fmol/cell) for non-toxic species *A. insuetum* and *P. triestinum* S32
Optimization of Chromabond^R HILIC SPE conditions using the non-labelled standard S33
Optimized sample clean-up for HR HILIC-ESI-Q-tof-MS and MS/MS S34
The linearity of modified column-switching HR HILIC-ESI-Q-tof-MS S35
Validation by the highly labelled sample mixed with the non-labelled standard S36
Calculation of peak area removing the contribution of the naturally occurring stable isotopes S37
Quantitation of the biosynthetic intermediates, the shunt product, and STXs in non-toxic and toxic dinoflagellates using column-switching HILIC- MS/MS (MRM) S38

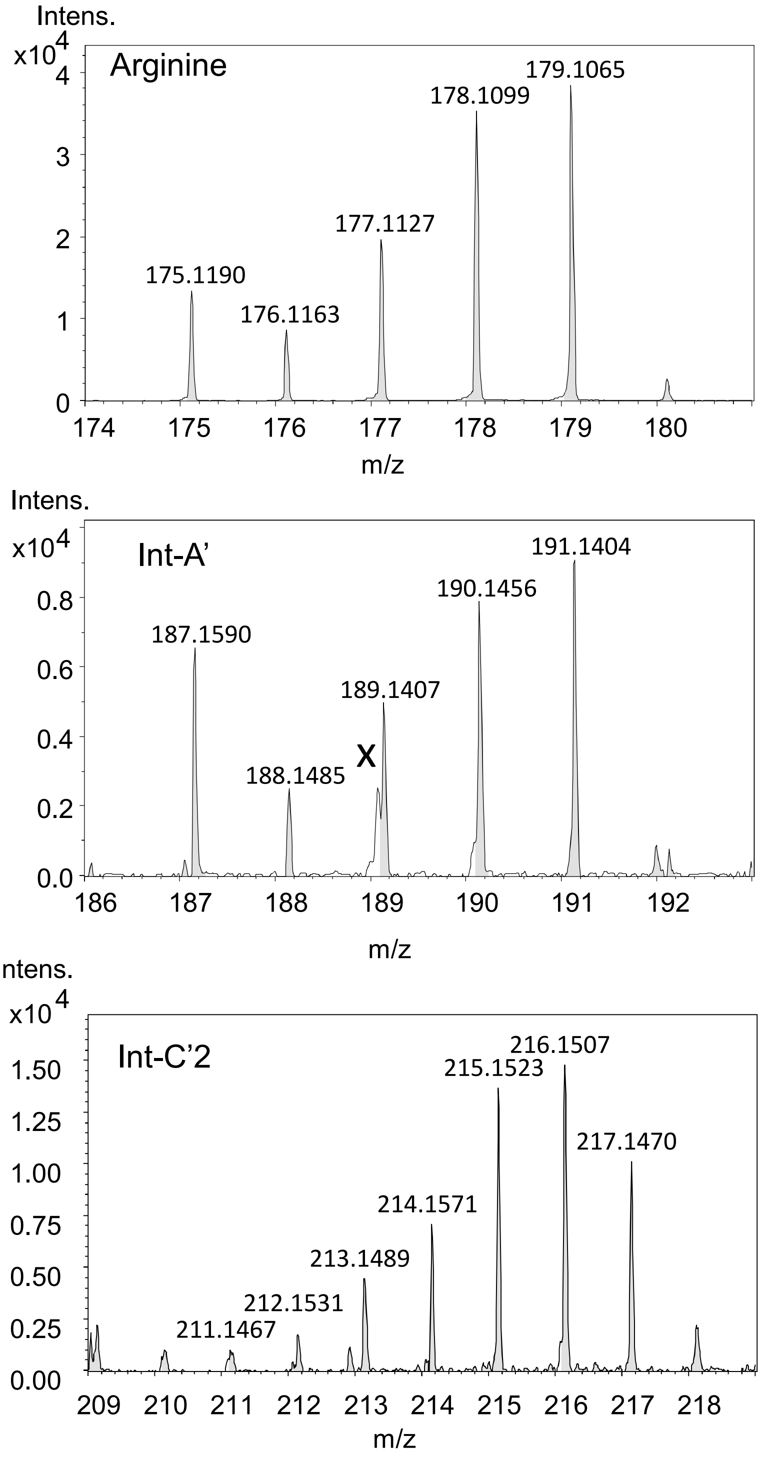

Figure S-1. Isotope patterns of the labelled biosynthetic intermediates (arginine, Int-A', and Int-C'2) on Day 10

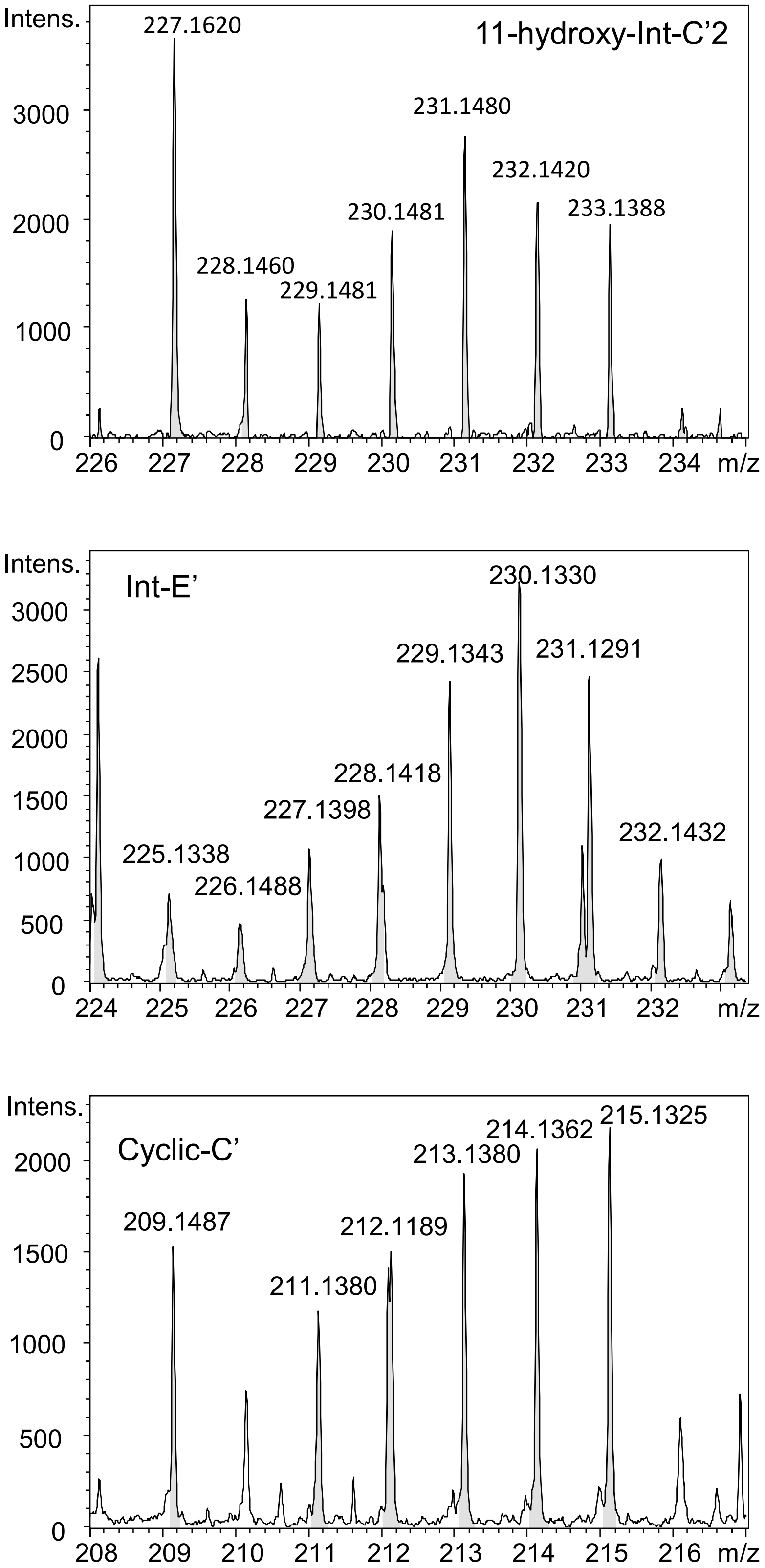

Figure S-2. Isotope patterns of the labelled biosynthetic intermediates (11-hydroxy-Int-C’2, Int-E’, and Cyclic-C’) on Day 10

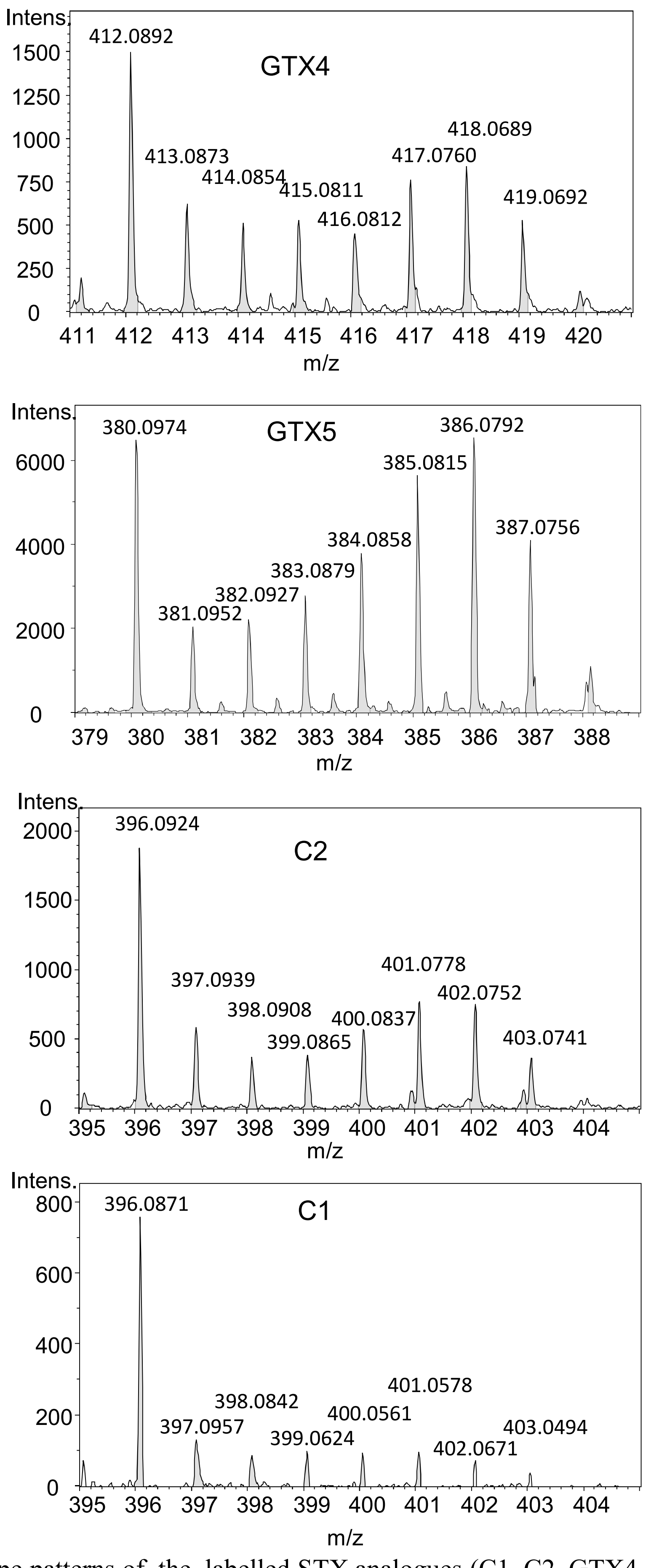

Figure S-3. Isotope patterns of the labelled STX analogues (C1, C2, GTX4, and GTX5) on Day 10

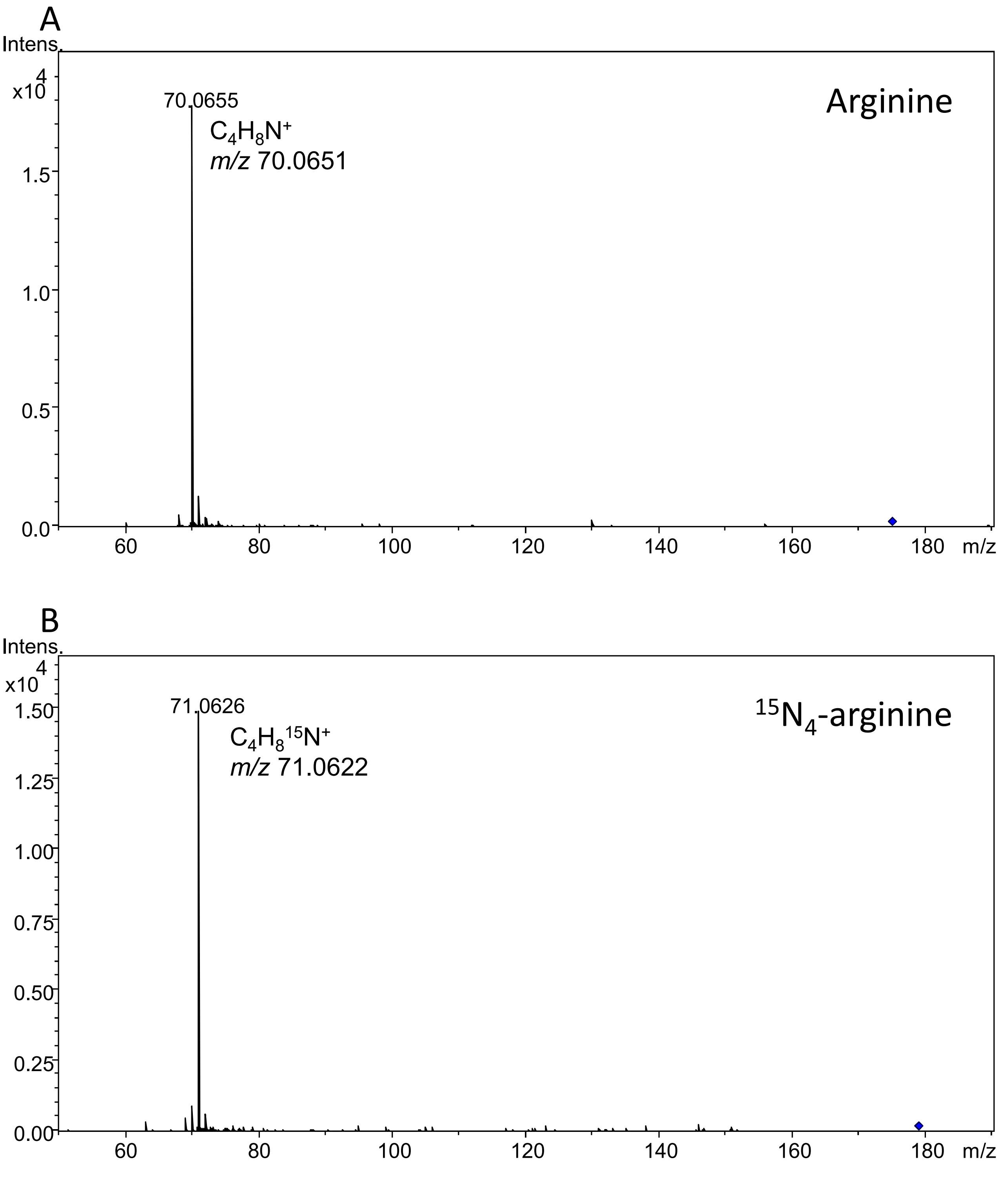

Figure S-4. MS/MS spectra of non-labelled (A) and perfectly labelled (B) arginine

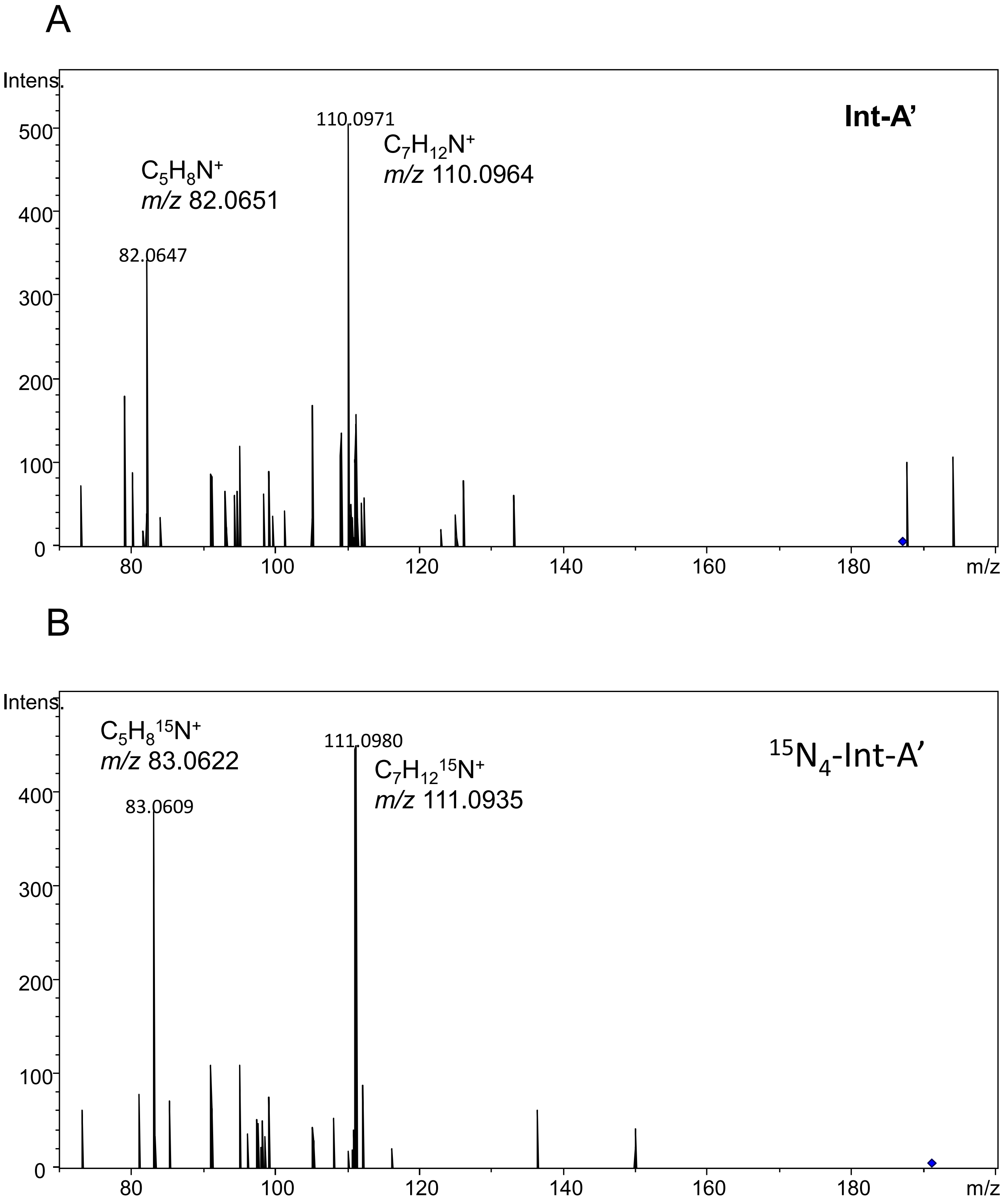

Figure S-5. MS/MS spectra of non-labelled (A) and perfectly labelled (B) Int-A'

A

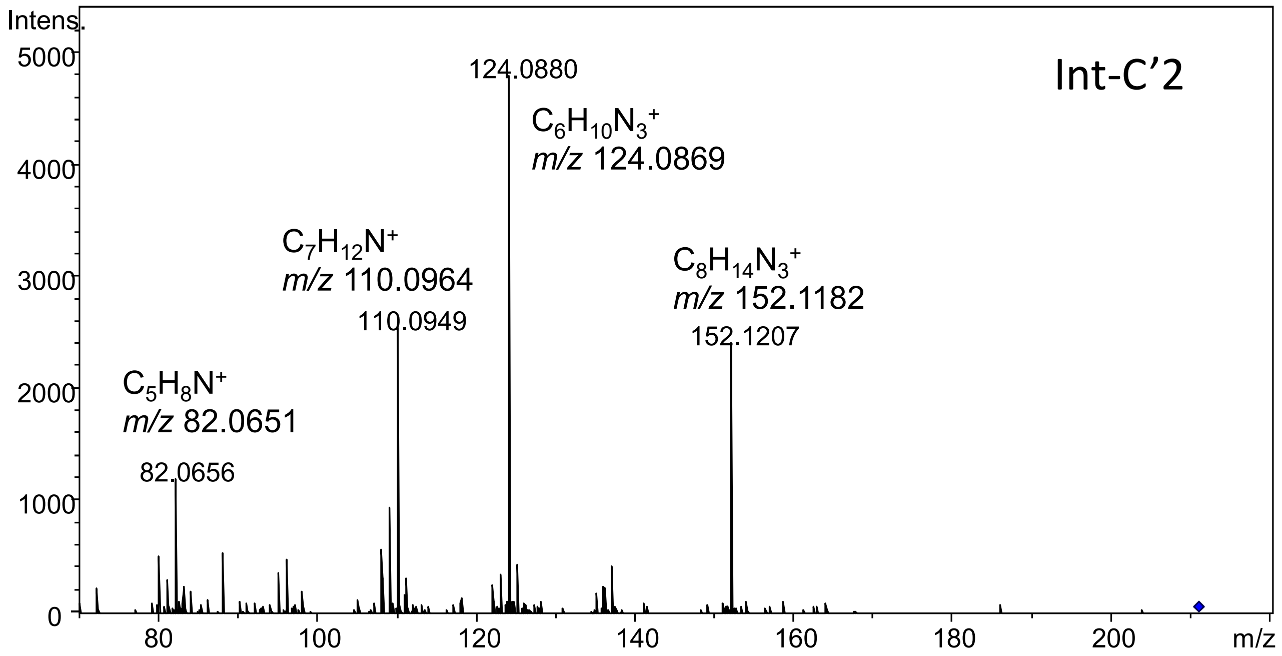

B

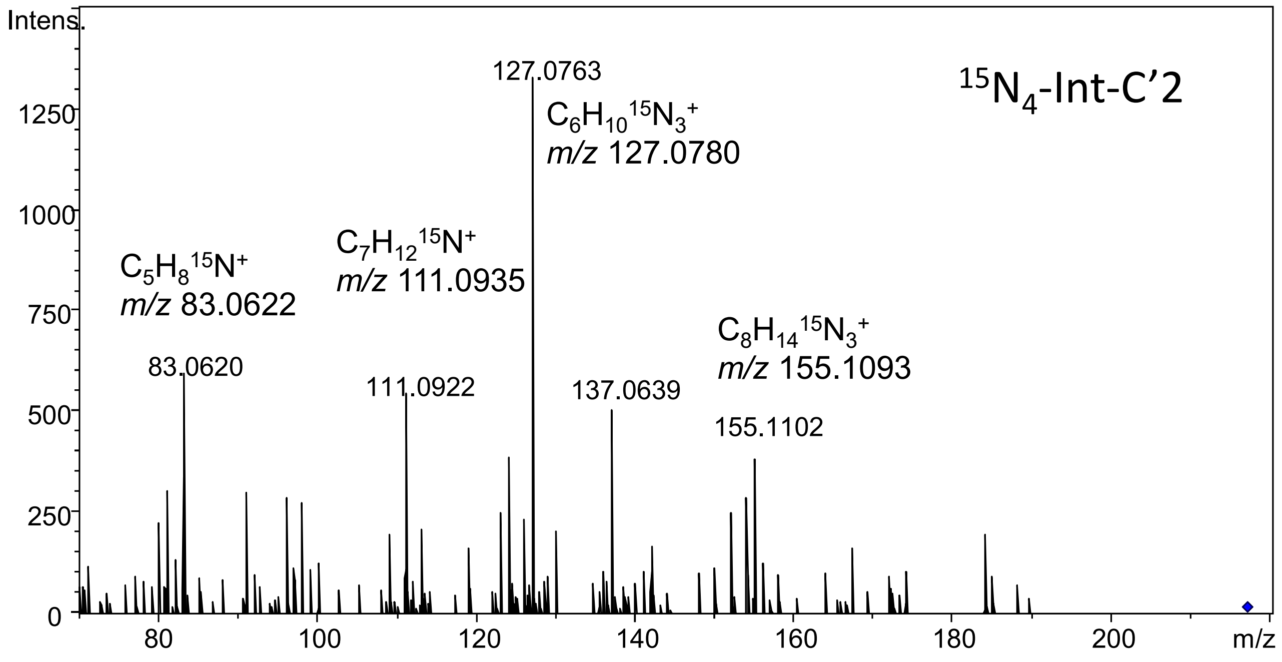

Figure S-6. MS/MS spectra of non-labelled (A) and perfectly labelled (B) Int-C’2

A

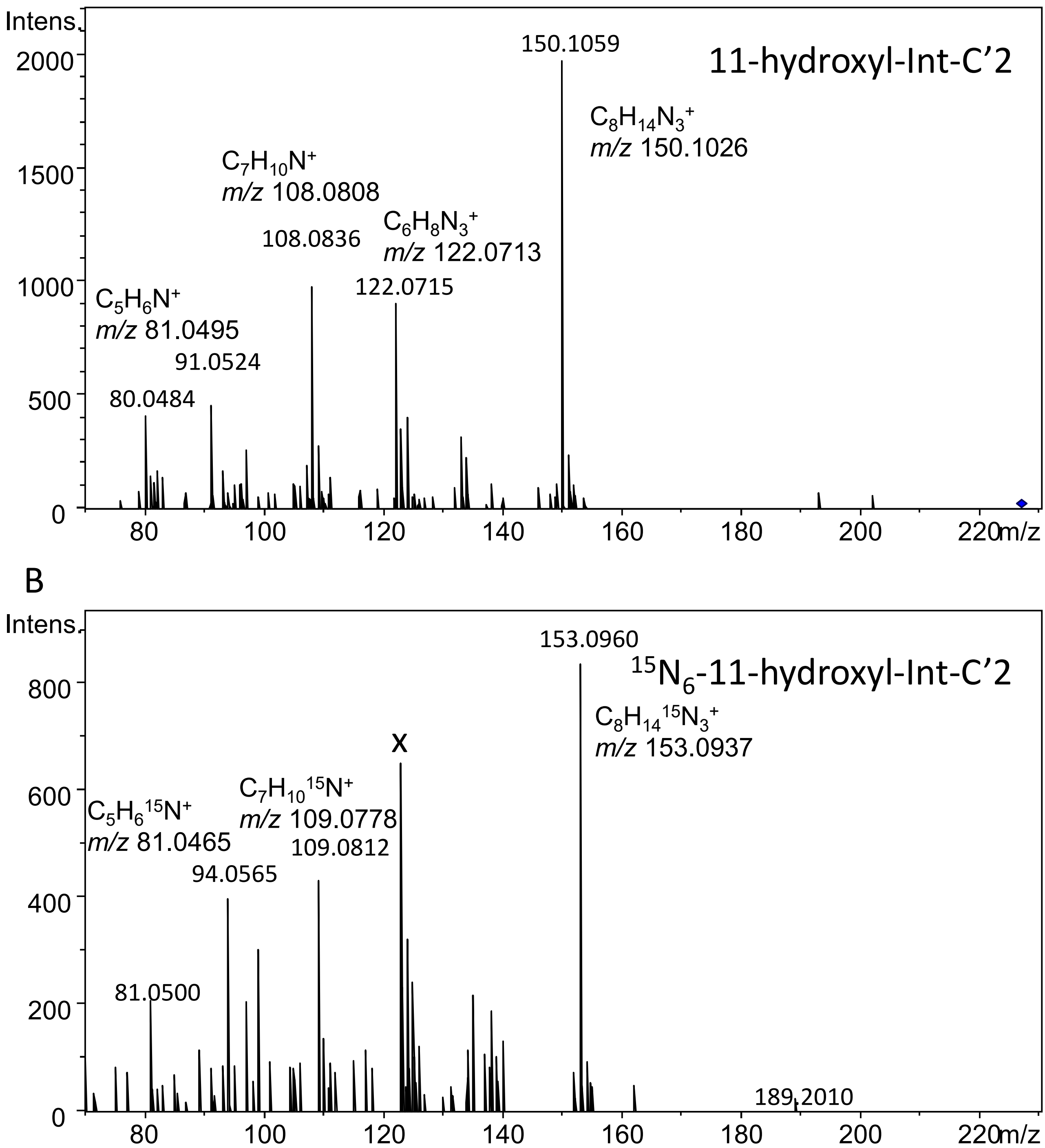

Figure S-7. MS/MS spectra of non-labelled (A) and perfectly labelled (B) 11-hydroxy-Int-C'2

A

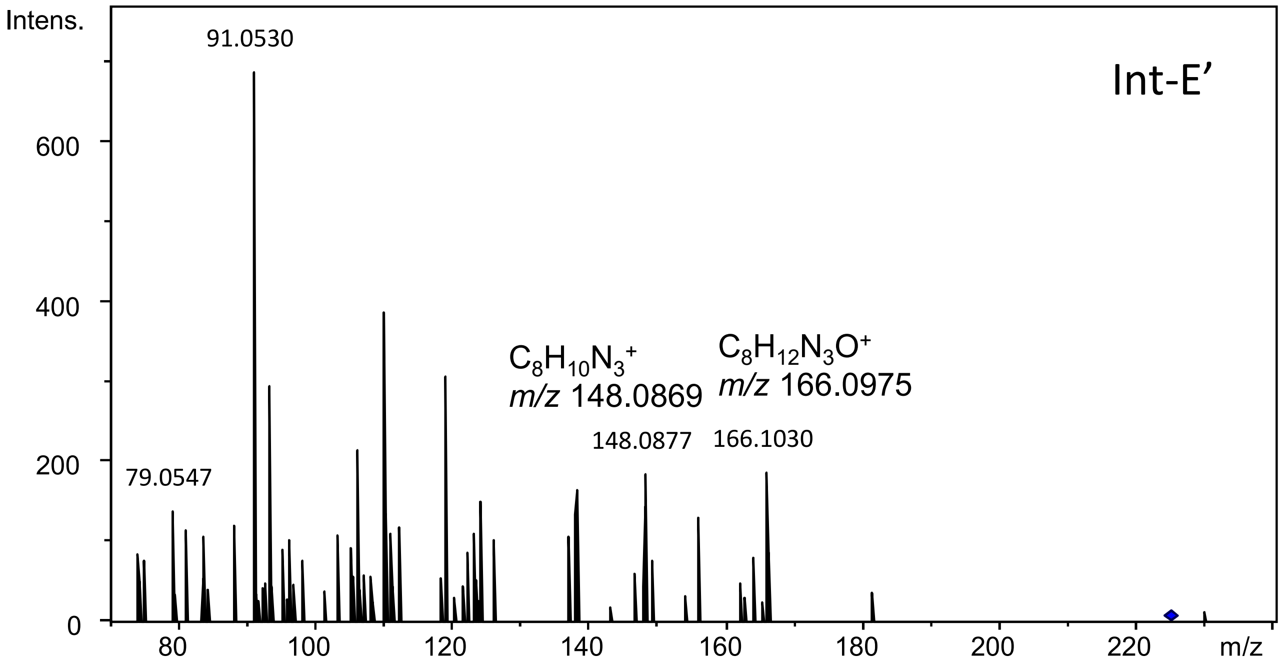

B

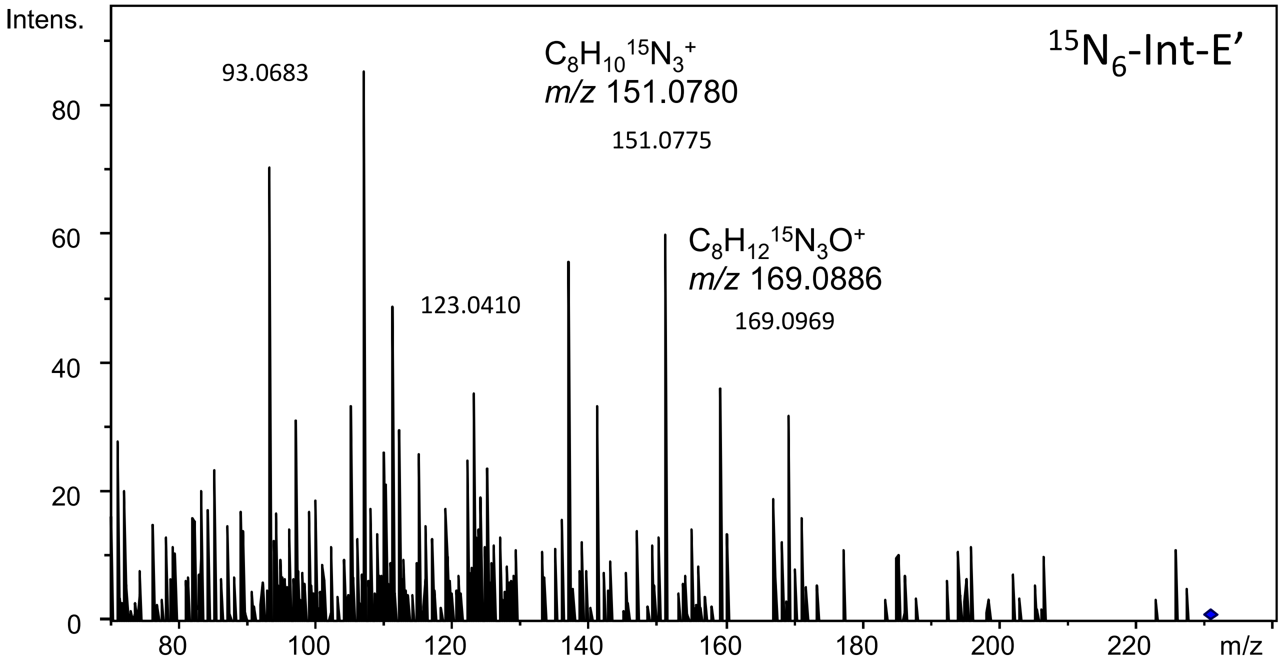

Figure S-8. MS/MS spectra of non-labelled (A) and perfectly labelled (B) Int-E'

A

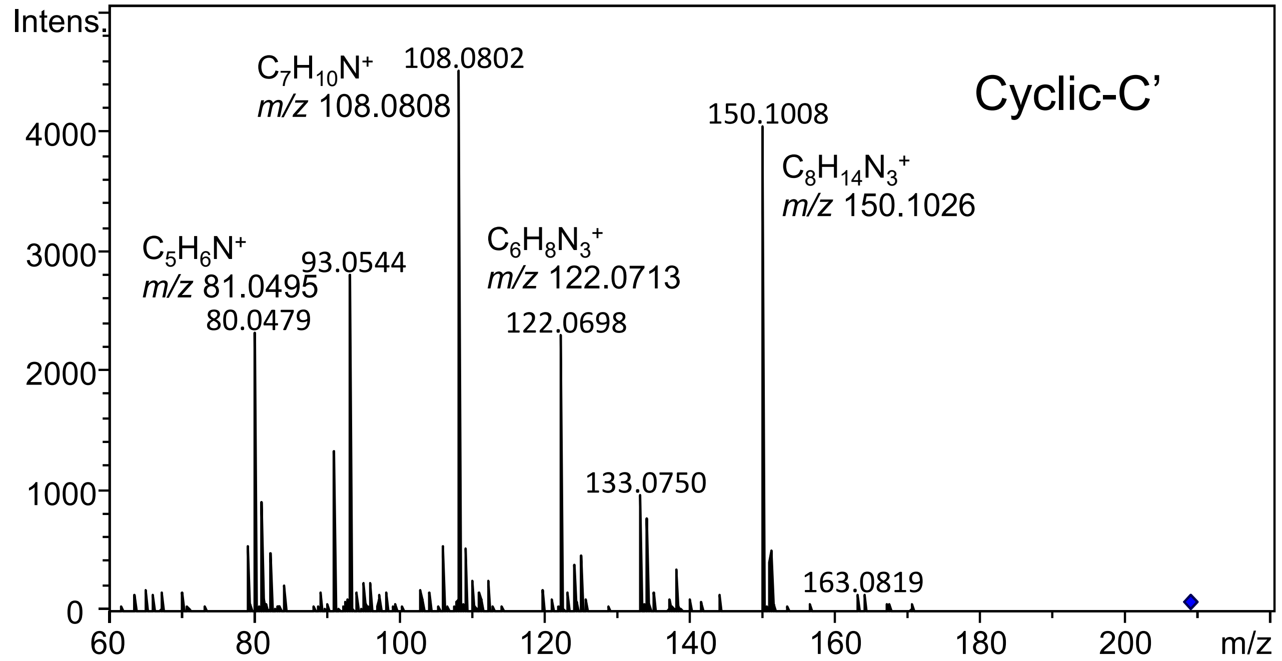

B

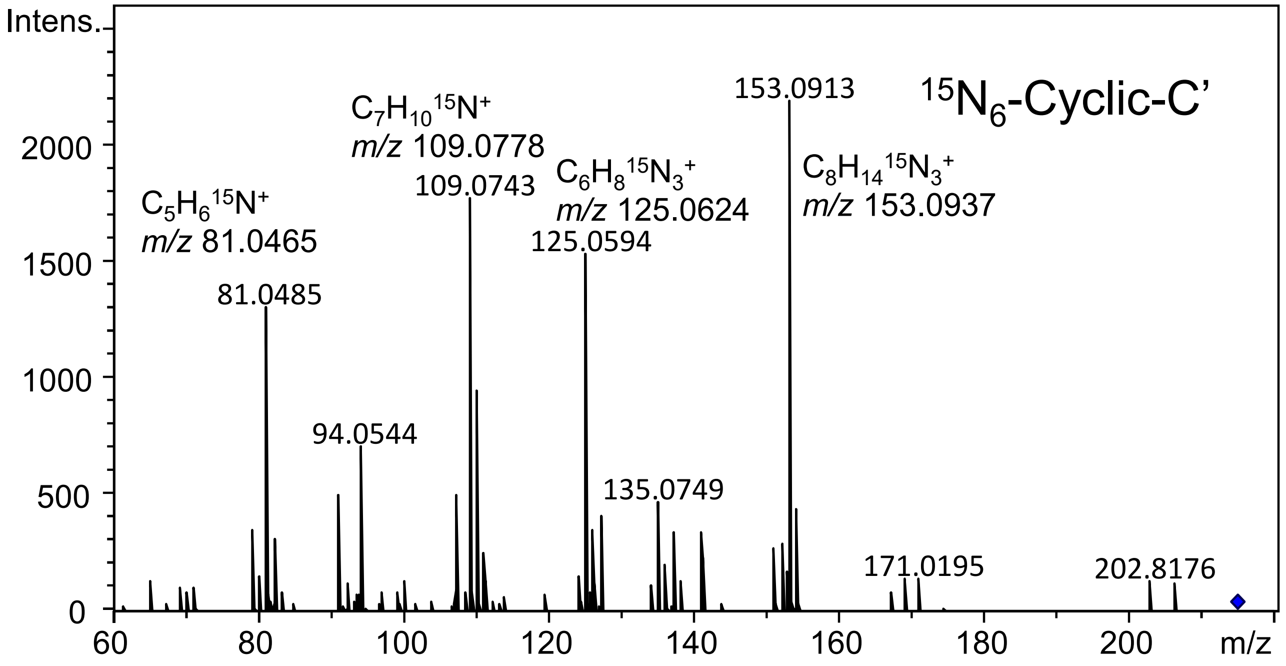

Figure S-9. MS/MS spectra of non-labelled (A) and perfectly labelled (B) Cyclic-C’

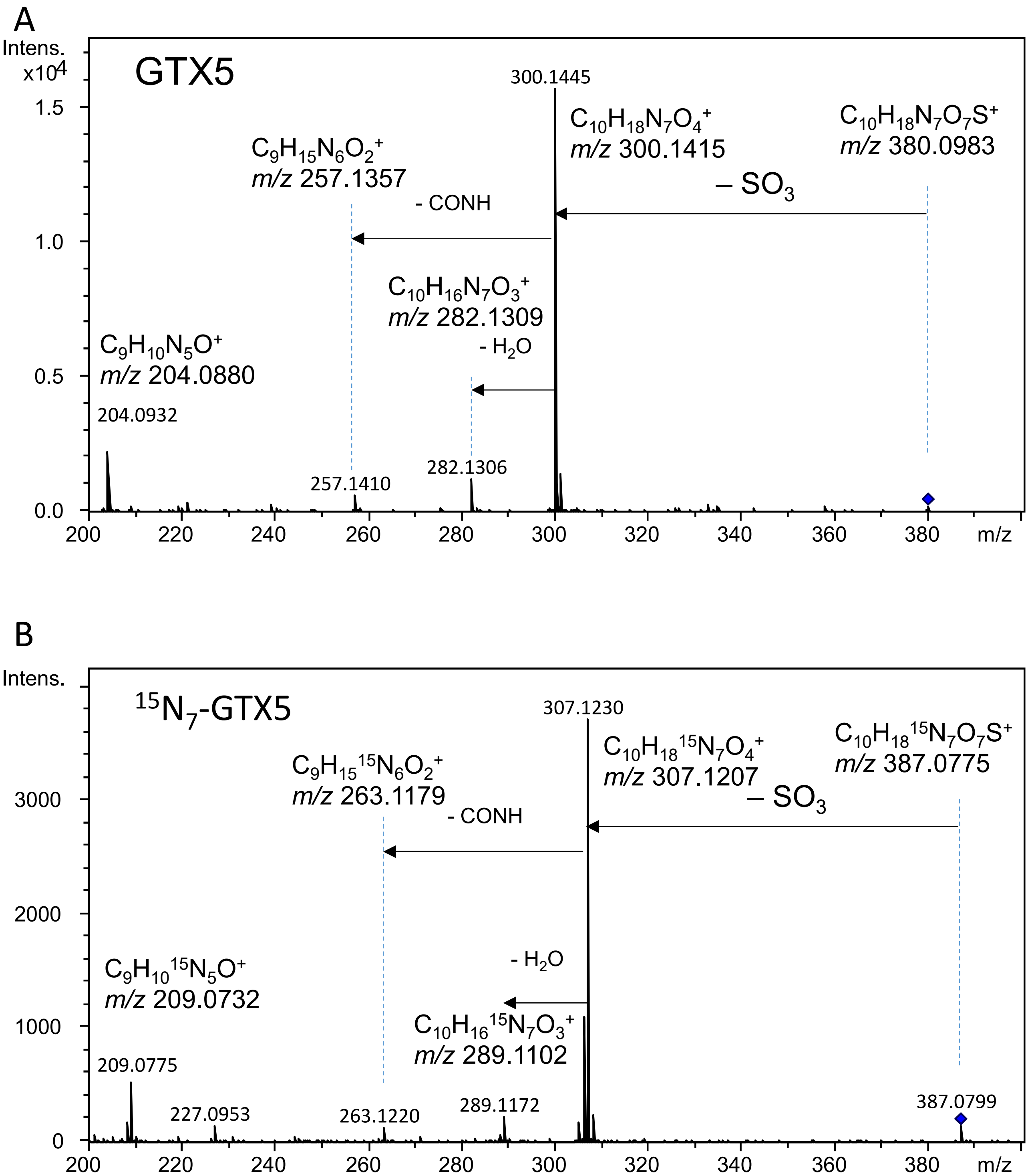

Figure S-10. MS/MS spectra of non-labelled (A) and perfectly labelled (B) GTX5

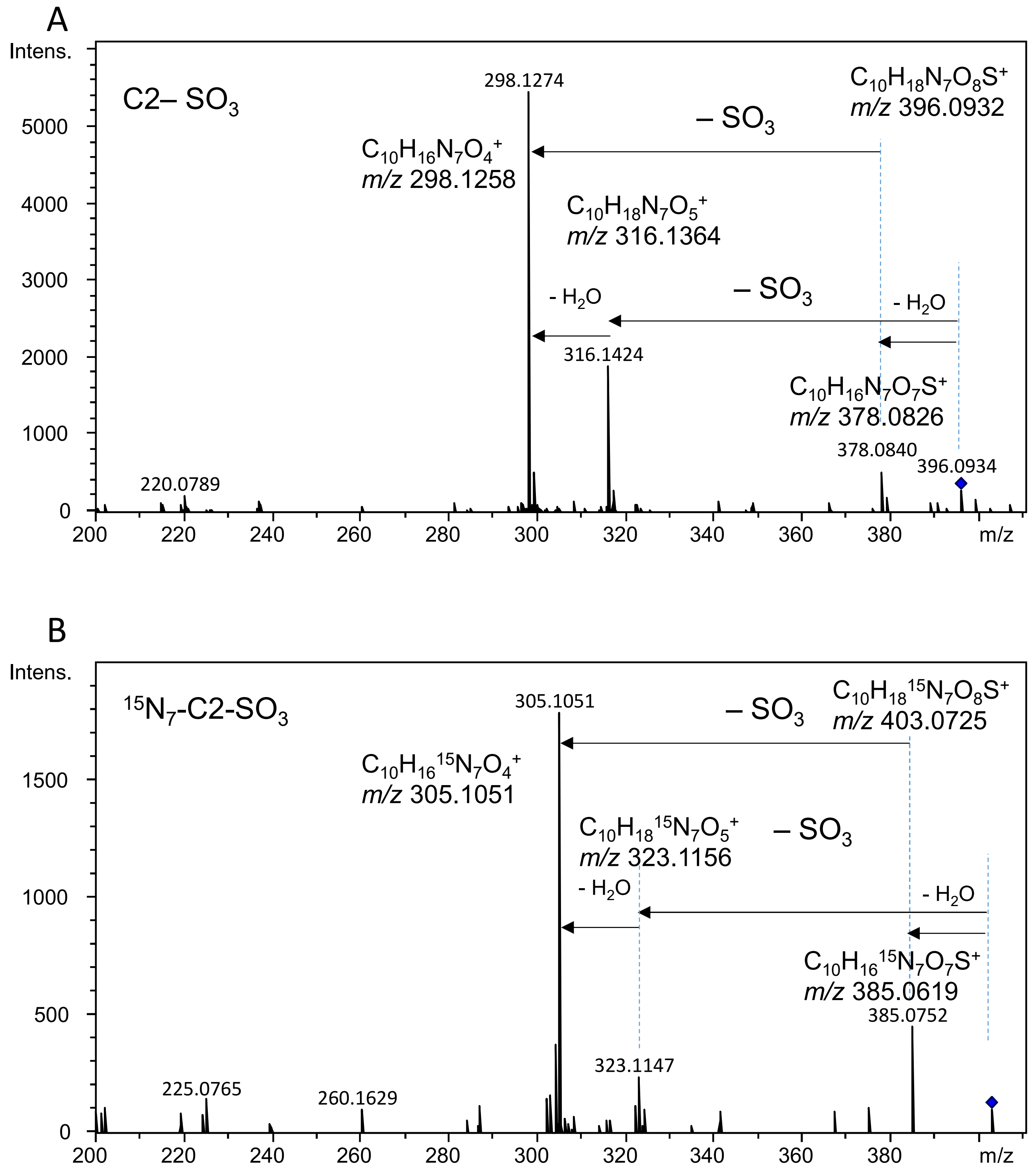

Figure S-11. MS/MS spectra of non-labelled (A) and perfectly labelled (B) C2

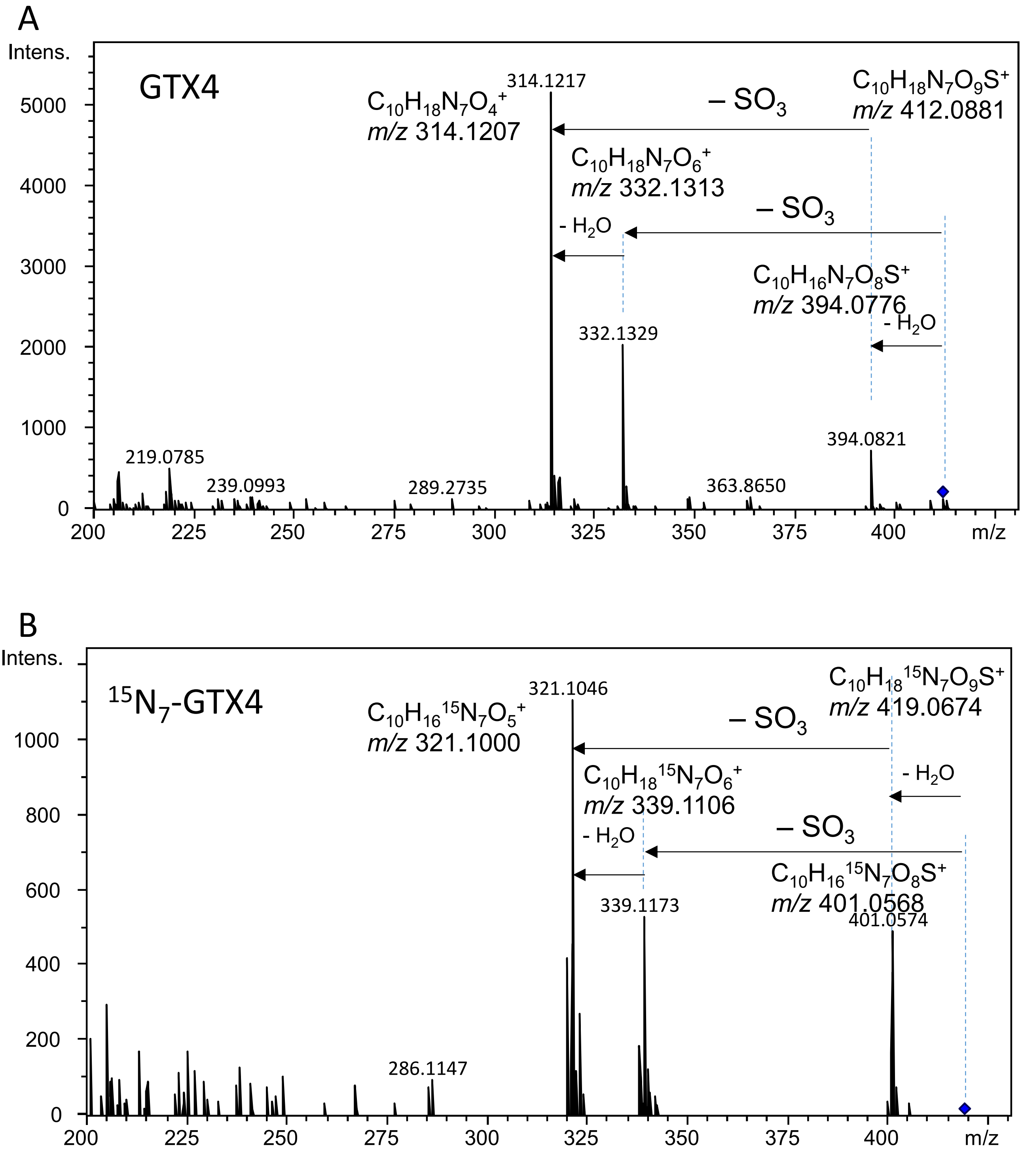

Figure S-12. MS/MS spectra of non-labelled (A) and perfectly labelled (B) GTX4

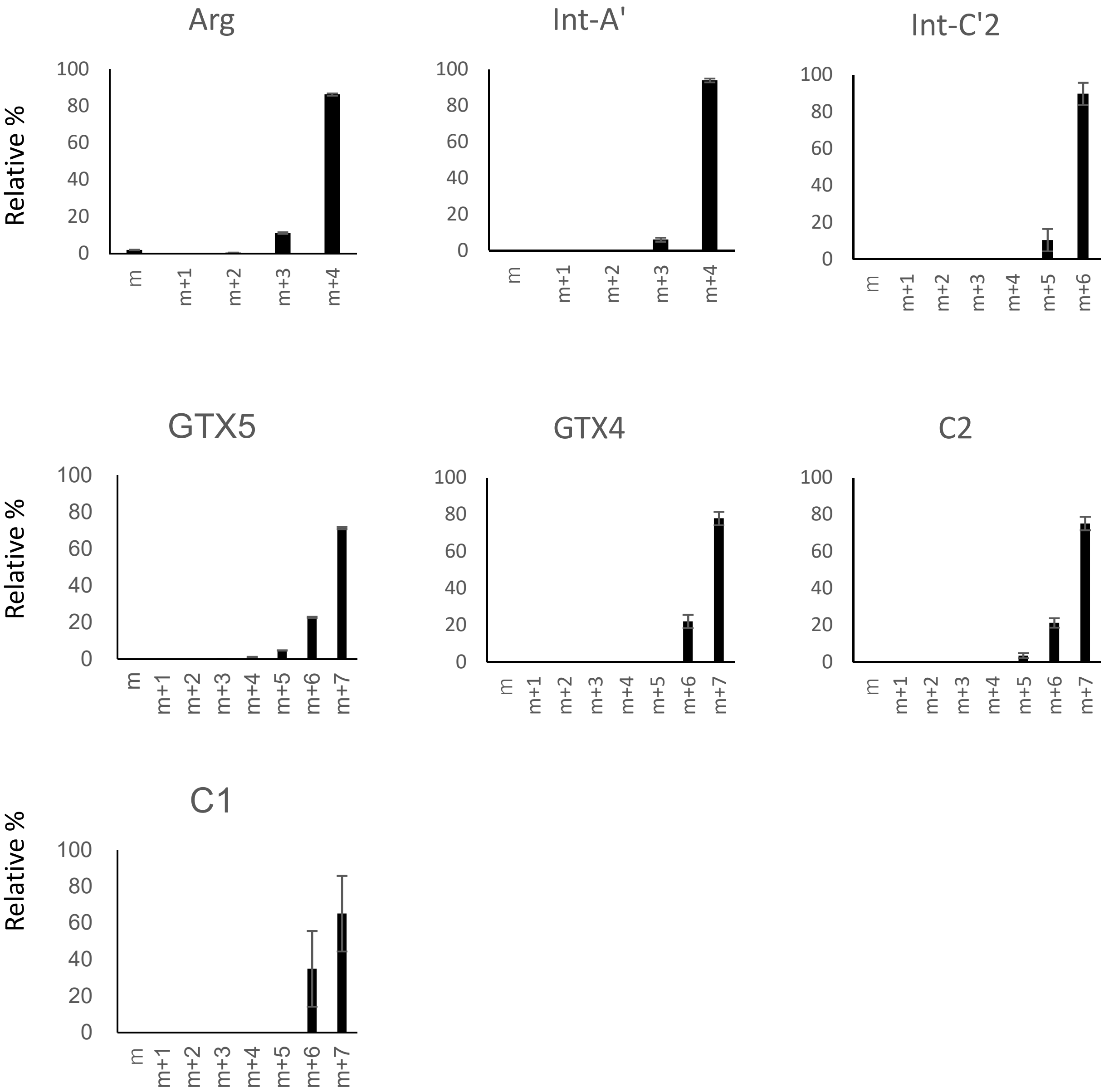

Figure S-13. Relative abundance % of each isotopomer of the biosynthetic intermediates in *A. catenella* after a 2-month passage in ^{15}N-NaNO$_3$ medium

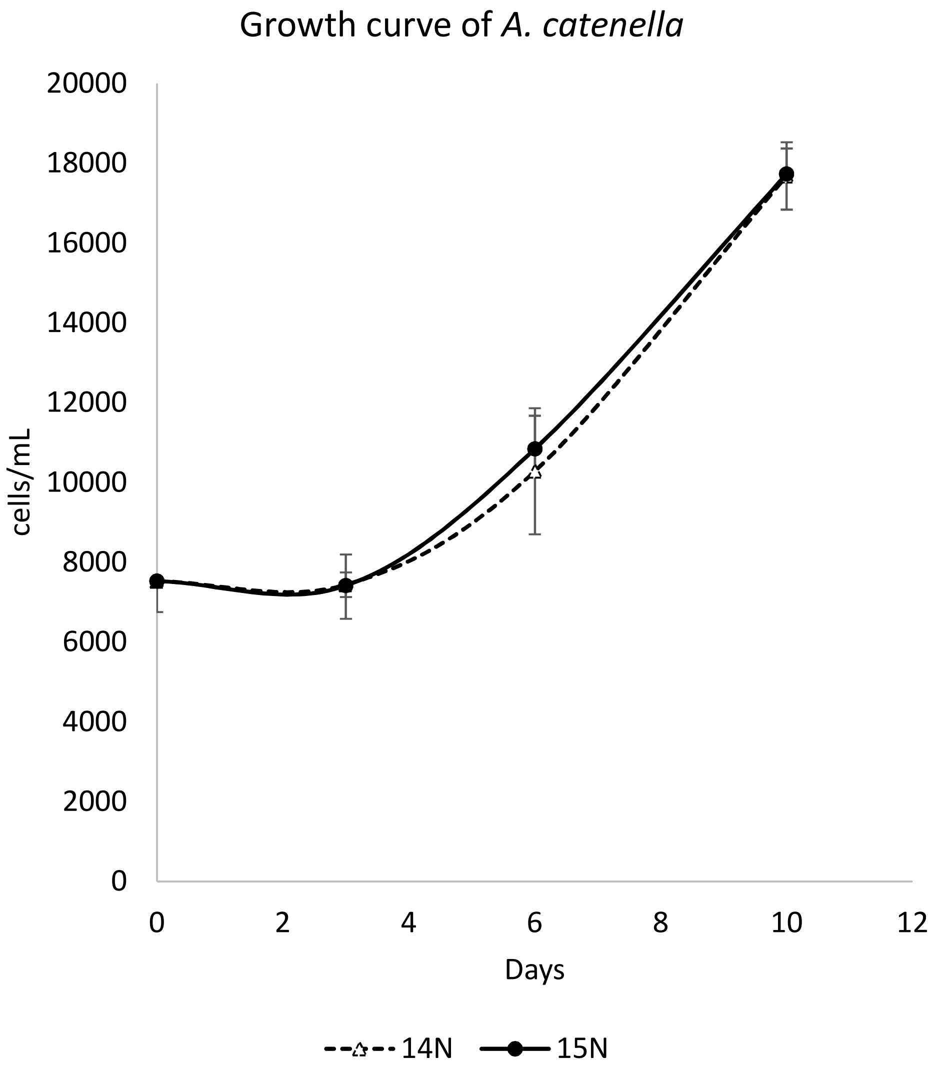

Figure S-14. Growth curve of *A. catenella* cultured with $^{15}N\text{-}NO_3$ or $^{14}N\text{-}NO_3$

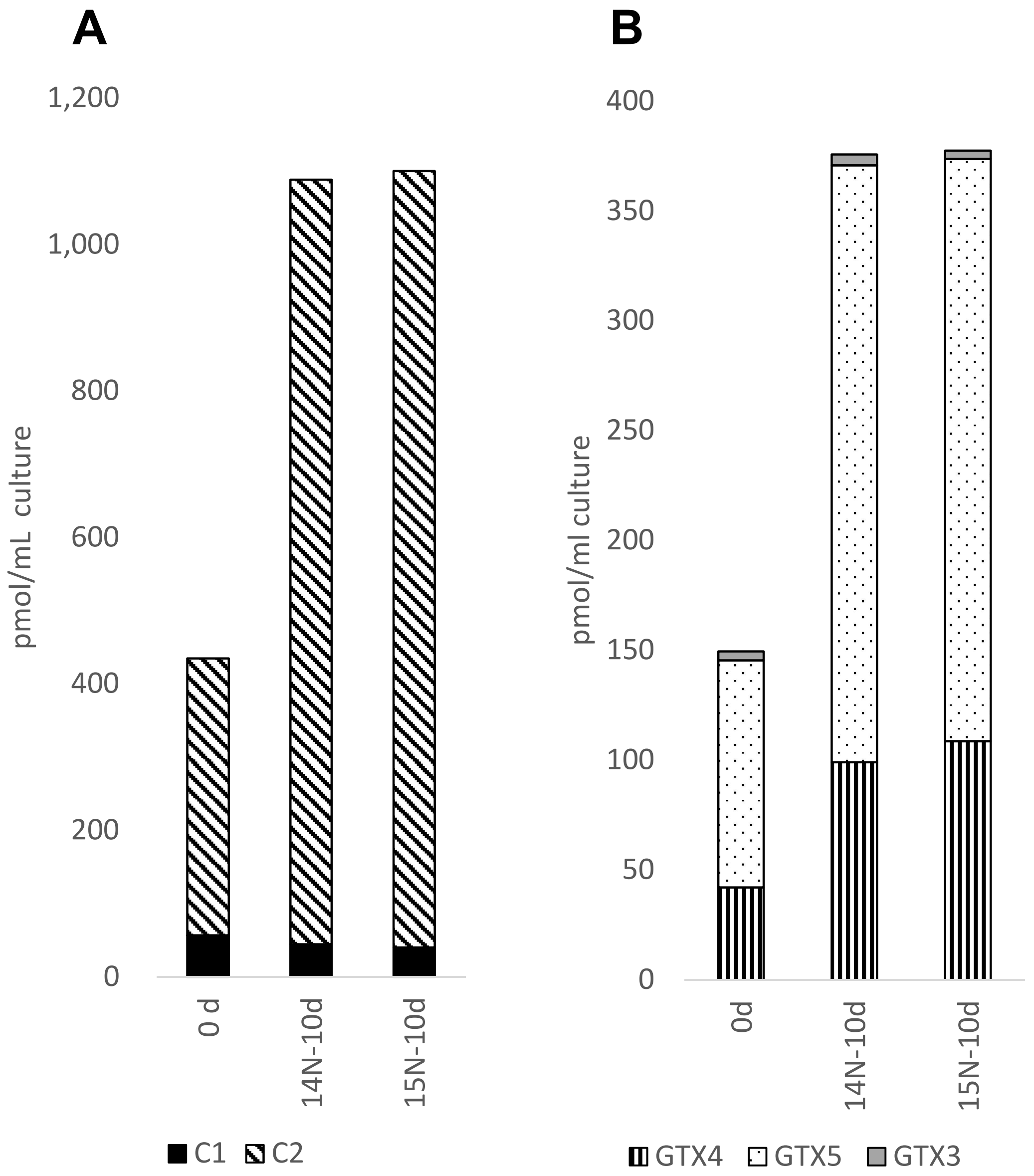

Figure S-15. Day-10 toxin contents of *A. catenella* cultured in ^{15}N-NaNO$_3$ or ^{14}N-NO$_3$. (A) C-toxins, (B) GTXs.

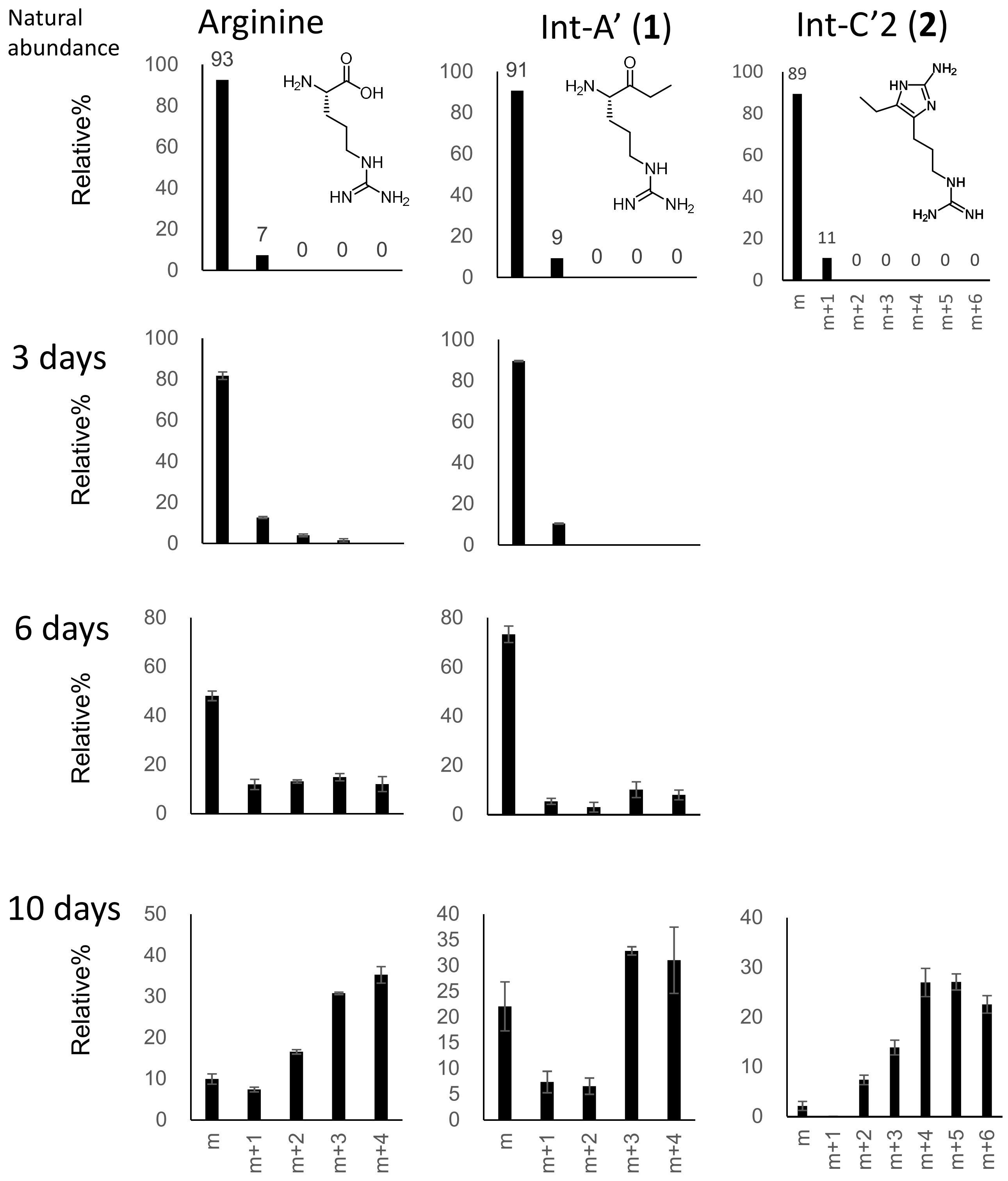

Figure S-16. Relative % of peak area of each isotopomer of the precursor and the biosynthetic intermediates at 3, 6, and 10 days after the addition of ^{15}N-NO_3 medium to *A. catenella*
Data are presented as mean ± SD (n=3).

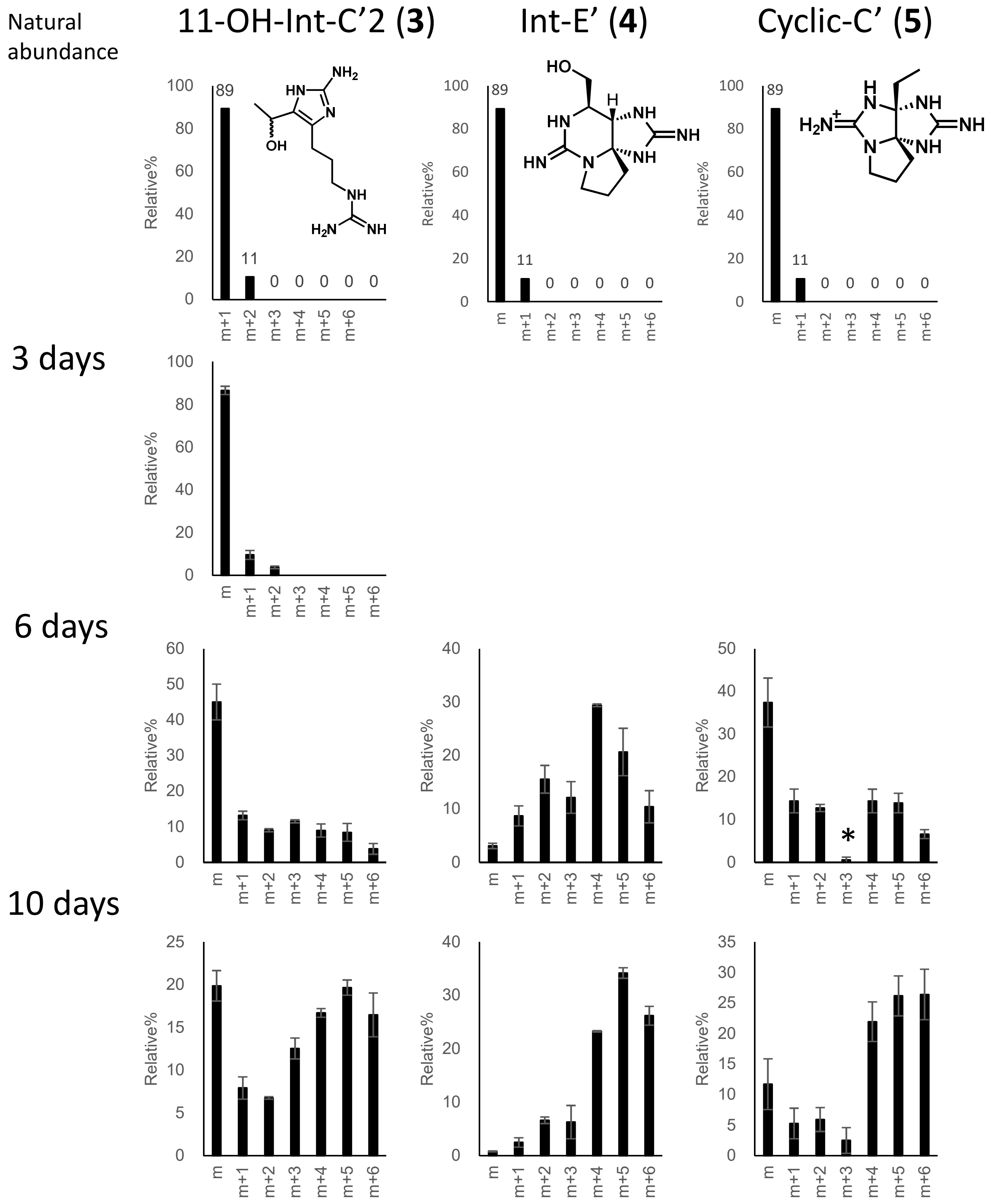

Figure S-17. Relative % of peak area of each isotopomer the biosynthetic intermediates, 11-hydroxyl-Int-C’2 and Int-E’ at 3, 6, and 10 days after the addition of $^{15}N-NO_3$ medium to *A. catenella*
Data are presented as mean ± SD (n=3). * This isotopomer was suppressed by matrix.

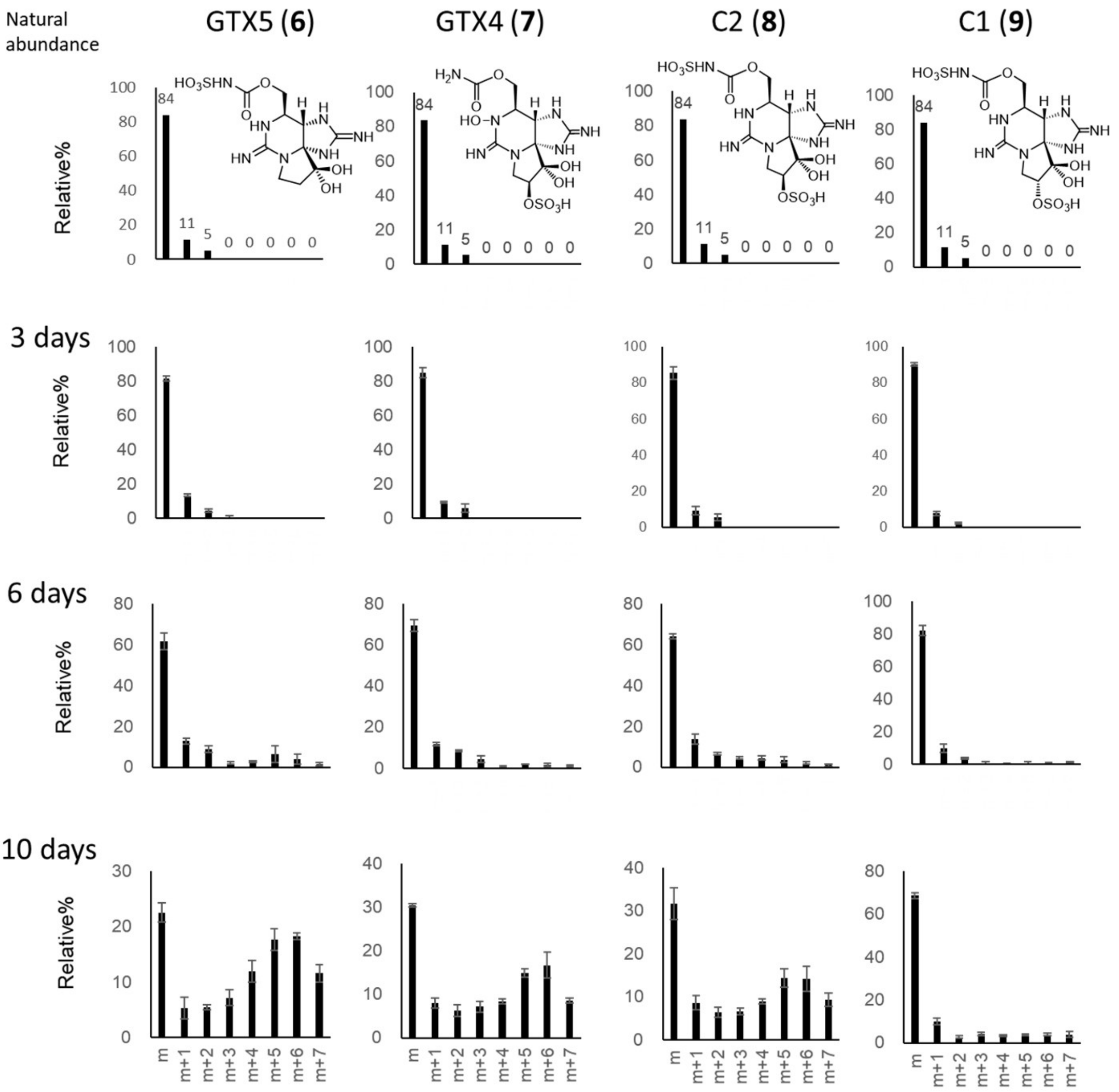

Figure S-18. Relative % of peak of each isotopomer of the STXs at 3, 6, and 10 days after the addition of ^{15}N-NO_3 medium in *A. catenella*
Data are presented as mean ± SD (n=3).

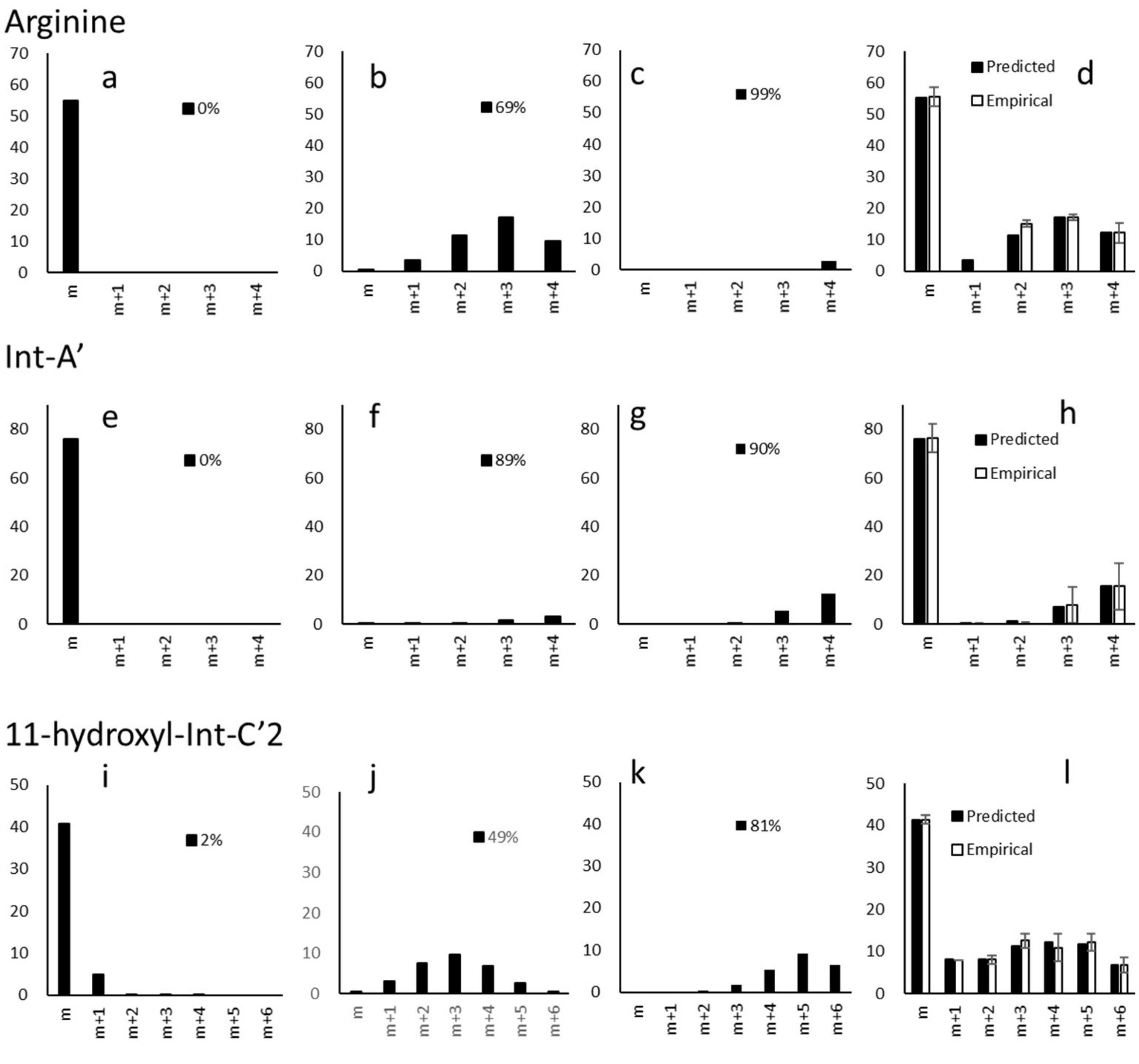

Figure S-19. Three populations stochastically predicted by empirical relative % of each isotopomer on Day 6 Arginine (a, b, c), Int-A' (e, f, g) and 11-hydroxyl-Int-C'2 (i, j, k), hypothesizing the binominal distribution and the merged graph (d, h and l) of predicted data and empirical data

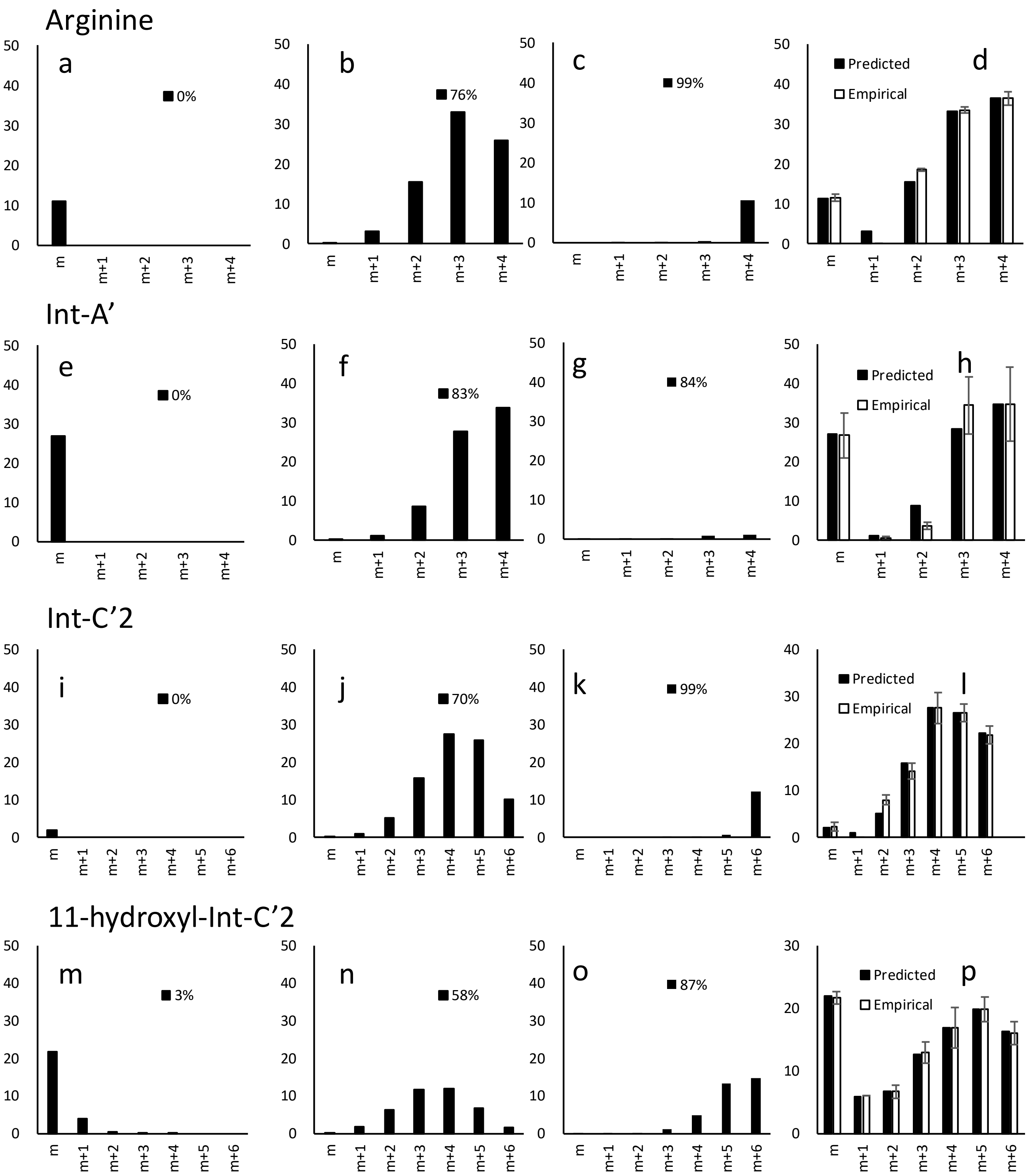

Figure S-20. Three populations stochastically predicted by empirical relative % of each isotopomer on Day 10 Arginine (a, b, c), Int-A’ (e, f, g), Int-C’2 (i, j, k) and 11-hydroxyl-Int-C’2 (m, n, o), hypothesizing the binominal distribution and the merged graph (d, h, l and p) of predicted data and empirical data

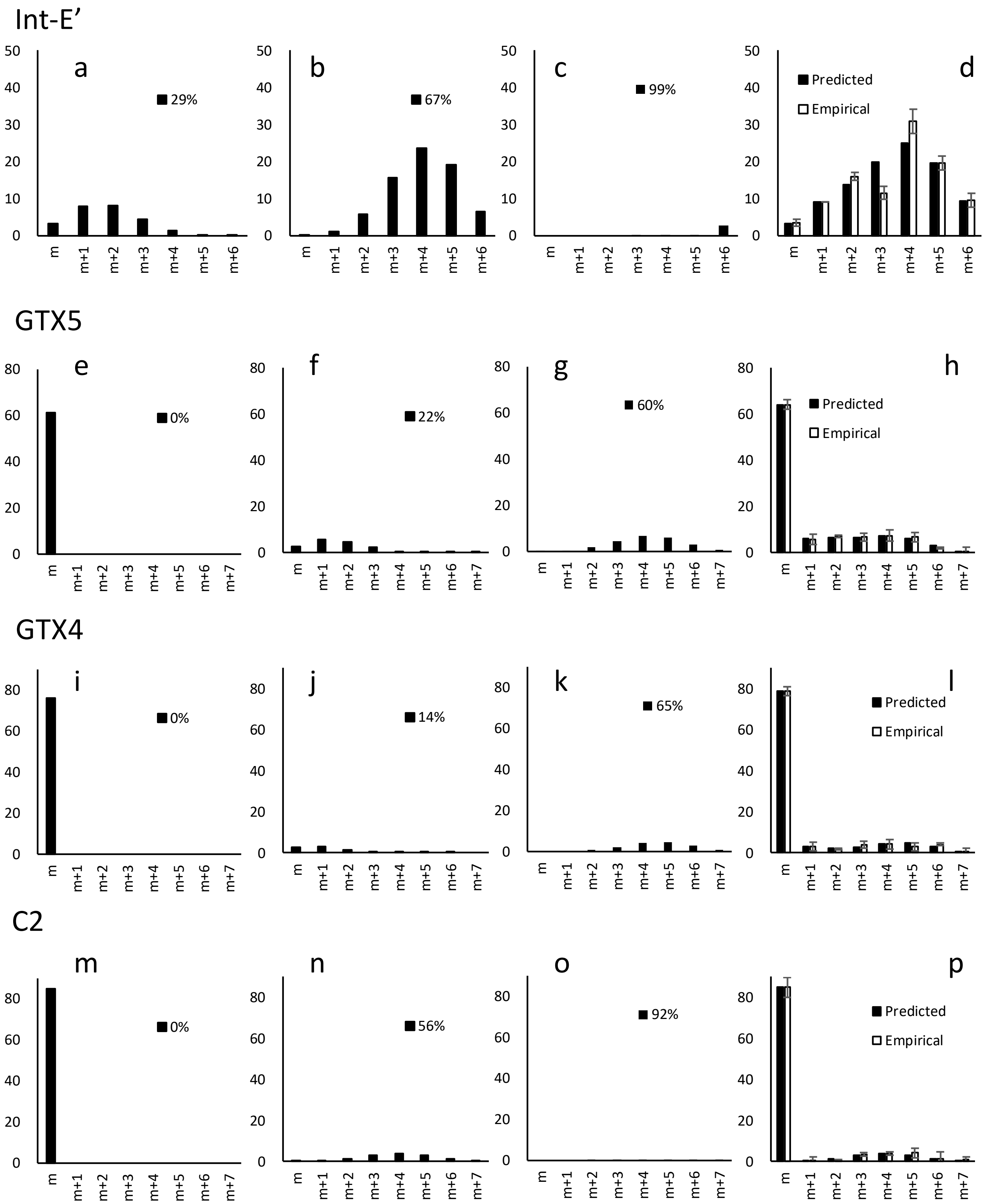

Figure S-21. Three populations stochastically predicted by empirical relative % of each isotopomer on Day 6
Int-E’ (a, b, c), GTX5(e, f, g), GTX4 (i, j, k) and C2 (m, n, o), hypothesizing the binominal distribution and the merged graph (d, h, l and p) of predicted data and empirical data

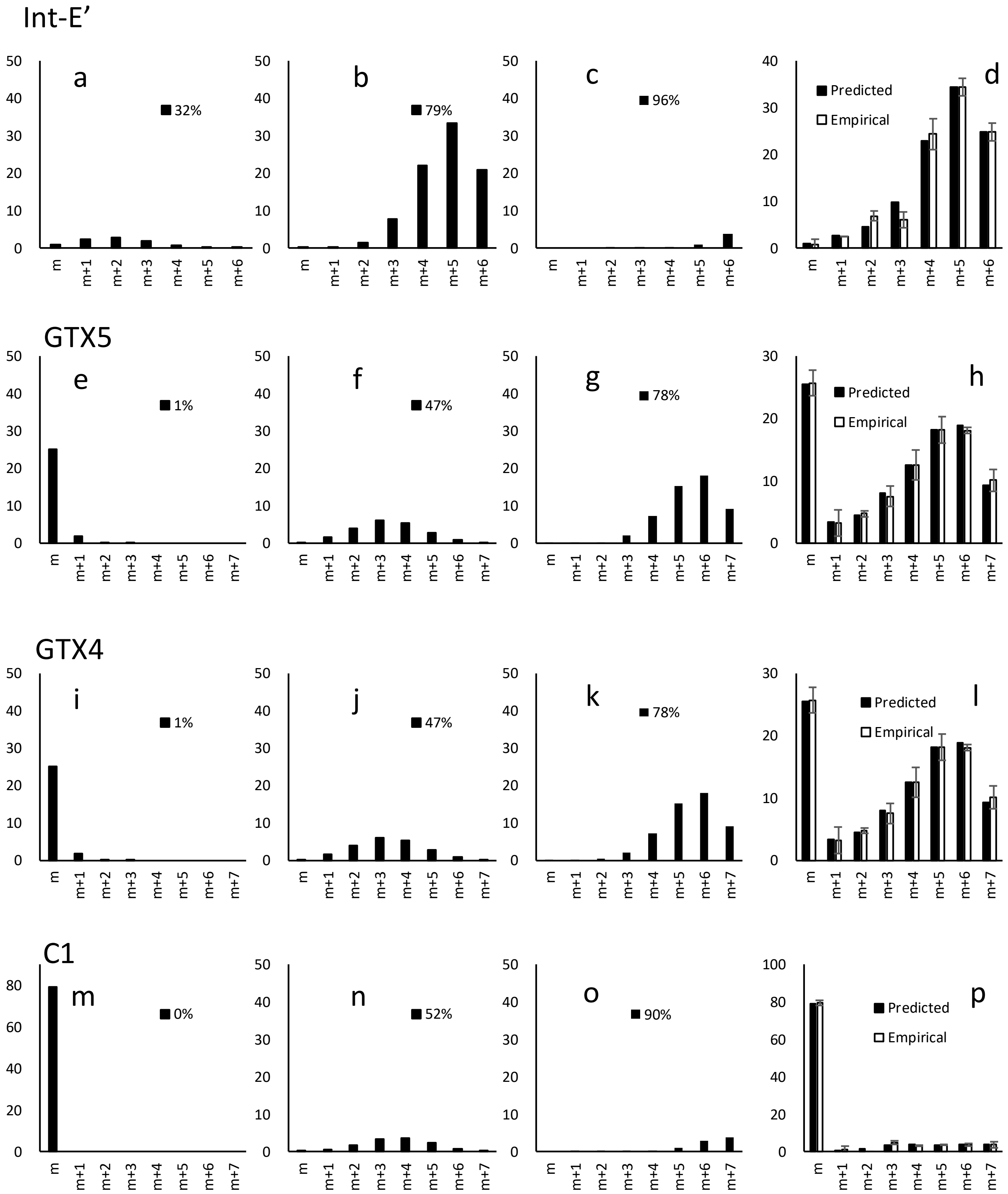

Figure S-22. Three populations stochastically predicted by empirical relative % of each isotopomer on Day 10

Int-E' (a, b, c), GTX5(e, f, g), GTX4 (i, j, k) and C1 (m, n, o), hypothesizing the binominal distribution and the merged graph (d, h, l and p) of predicted data and empirical data

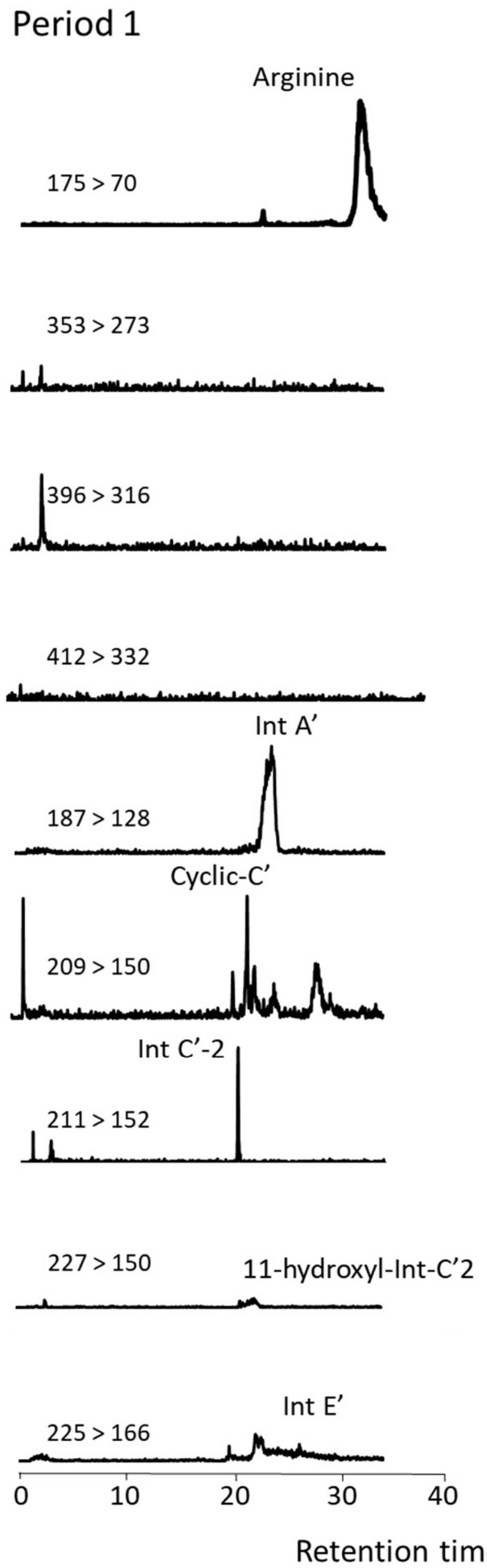

Figure S-23. HILIC-MS-MRM chromatogram of toxic *G. catenatum* extract

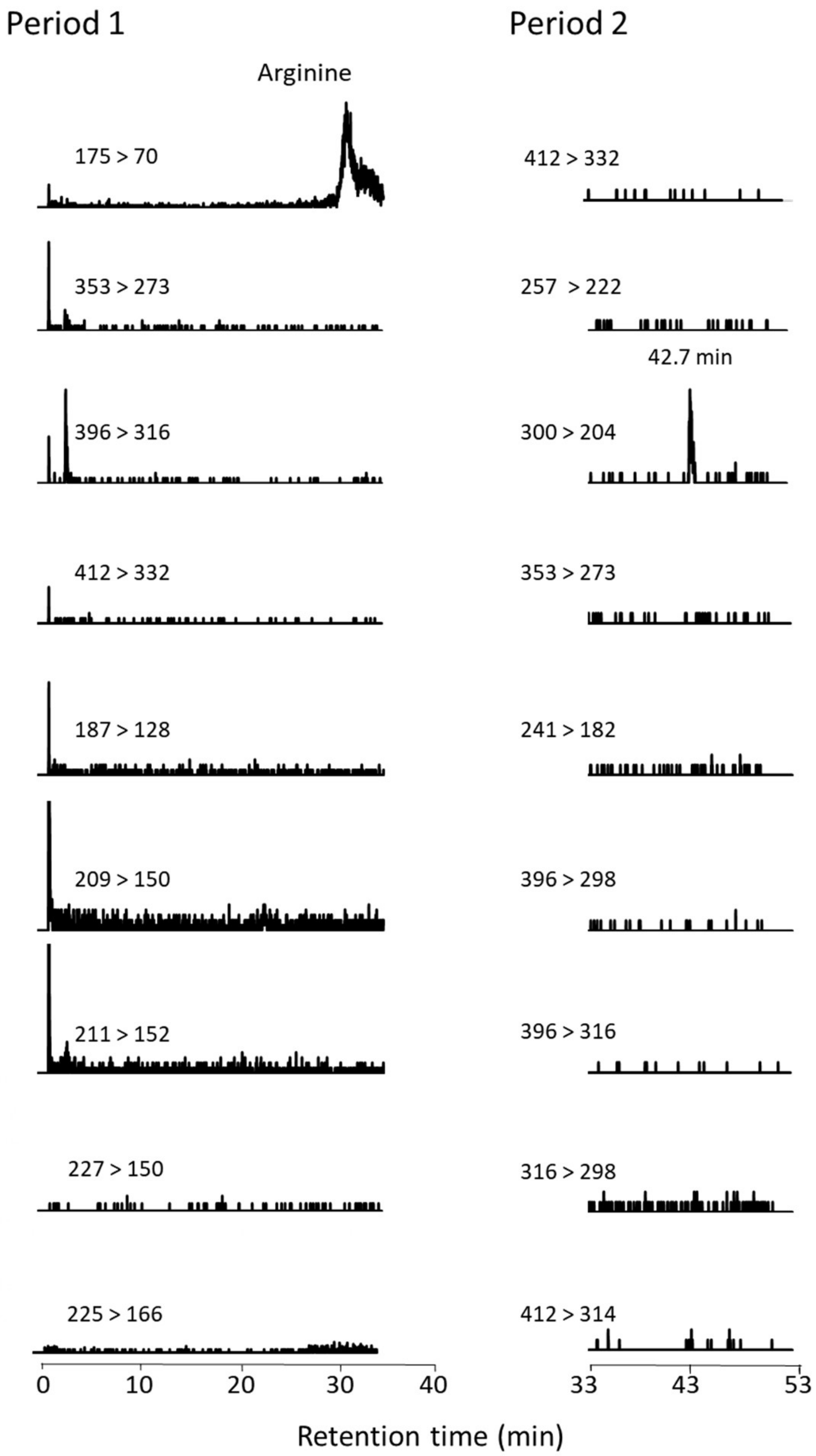

Figure S-24. HILIC-MS-MRM chromatogram of non-toxic *A. insuetum* extract

Table S-1.
The theoretical values for the isotopomers with different numbers of ^{15}N

Number of ^{15}N	0	1	2	3	4	5	6	7
Arginine	175.1190	176.1160	177.1130	178.1101	179.1071			
Int-A'	187.1553	188.1524	189.1494	190.1464	191.1435			
Int-C'2	211.1666	212.1636	213.1606	214.1577	215.1547	216.1517	217.1488	
11-hydroxy-Int-C'2	227.1615	228.1585	229.1556	230.1526	231.1496	232.1467	233.1437	
Int-E'	225.1458	226.1429	227.1399	228.1369	229.1340	230.1310	231.1280	
Cyclic-C'	209.1509	210.1480	211.1450	212.1420	213.1391	214.1361	215.1331	
C2-SO_3	396.0932	397.0902	398.0873	399.0843	400.0813	401.0784	402.0754	403.0725
GTX5	380.0983	381.0953	382.0924	383.0894	384.0864	385.0835	386.0851	387.0775
GTX4	412.0881	413.0852	414.0822	415.0792	416.0763	417.0733	418.0703	419.0674

C1 was analyzed by the same theoretical values as for C2.

Table S-2. Clean up conditions employed for Chromabond^{R} HILIC and the resulting recovered compounds

Entry #	Conditioning	Washing	Elution	Recovered compounds
1	CH_3CN-H_2O with 0.1% HCOOH = 8:2	CH_3CN-H_2O with 0.1% HCOOH = 8:2	CH_3CN-H_2O with 0.1% HCOOH = 1:9	GTX1-5, STXs, C1, C2
2	THF	THF	0.5 M AcOH or 0.2M HCOOH	Arg, Int-A', IntC'2, Cyclic-C', GTX1-3, GTX5, STXs
3	THF	THF, CH_3CN, CH_3CN-H_2O with 0.1% HCOOH = 9:1	0.2 M HCOOH	Arg, Int-A', IntC'2, Cyclic-C', GTX1-5, STXs, C1, C2

Table S-3. Example of calculation of peak areas removing the contribution of the naturally occurring stable isotope

Peak area of each isotopomer of C2

m/z	396	397	398	399	400	401	402	403
Absolute peak area of C2	57129	10746	4704	9902	7942	25581	13239	12015
theoretical peak area (none of labelled 15N was introduced)	57129	7655	3485	0	0			
expected peak area after subtraction of nonlabelled C2	0	3091	1219	9902	7942			
theoretical peak area C2 involving one unit of labelled 15N		3091	433	189	0	0		
expected peak area after subtraction of C2 which involves 0 and 1 labelled 15N		0	786	9714	7942	25581		
theoretical peak area C2 involving 2 unit of labelled 15N			786	99	48	0	0	
expected peak area after subtraction of C2 which involves 0-2 labelled 15N			0	9615	7894	25581	13239	
theoretical peak area C2 involving 3 unit of labelled 15N				9615	1183	587	0	0
expected peak area after subtraction of C2 which involves 0-3 labelled 15N				0	6712	24994	13239	0
theoretical peak area C2 involving 4 unit of labelled 15N					6712	799	49	0
expected peak area after subtraction of C2 which involves 0-4 labelled 15N					0	24196	13190	12015
theoretical peak area C2 involving 5 unit of labelled 15N						24196	2613	1476
expected peak area after subtraction of C2 which involves 0-5 labelled 15N						0	10577	10539
theoretical peak area C2 involving 6 unit of labelled 15N							10577	1142
expected peak area after subtraction of C2 which involves 0-6 labelled 15N							0	9397
theoretical peak area C2 involving 7 unit of labelled 15N								9397

Natural Abundance (%)	M	M+1	M+2	M+3	M+4	M+5	M+6	M+7
non-labelled	100	13.4	6.1					
15N		100	14	6.1				
15N2			100	12.6	6.1			
15N3				100	12.3	6.1		
15N4					100	11.9	6.1	
15N5						100	10.8	6.1
15N6							100	10.8
15N7								100

number of incorporated 15N	0	1	2	3	4	5	6	7
Peak areas after removing those of the naturally occurring stable isotope	57129	3091	786	9615	6712	24196	10577	9397

Table S-4. Production rate (nM/d)

Day	Arginine	Int-A'	Int-C'2	Cyclic-C'	11-OH-Int-C'2	Int-E'	GTX5	GTX4	C2	C1
3	-162	0	ND	ND	-7	ND	-5	-4	10	-31
6	73	8	ND	12	15	15	42	9	136	-14
10	75	4	7	5	3	9	13	14	61	-6

ND: not determined

Table S-5. ^{15}N incorporation and their ratio calculated by three-population model for the isotopomer distribution % of Day 6 and Day 10

Arginine 6d

	15N %		Ratio
p	99%	A	3
q	69%	B	42
r	0%	C	55

Int-E' 6d

	15N %		Ratio
p	99%	A	3
q	67%	B	72
r	29%	C	25

Arginine 10d

	15N %		Ratio
p	99%	A	11
q	76%	B	78
r	0%	C	11

Int-E' 10d

	15N %		Ratio
p	96%	A	5
q	79%	B	86
r	32%	C	9

Int-A' 6d

	15N %		Ratio
p	90%	A	19
q	89%	B	5
r	0%	C	76

GTX5 6d

	15N %		Ratio
p	60%	A	23
q	22%	B	16
r	0%	C	61

Int-A' 10d

	15N %		Ratio
p	84%	A	2
q	83%	B	71
r	0%	C	27

GTX5 10d

	Incorporation %		Ratio
p	80%	A	47
q	53%	B	25
r	1%	C	28

Int-C'2

	15N %		Ratio
p		A	
q		B	
r		C	

GTX4 6d

	15N %		Ratio
p	65%	A	16
q	14%	B	8
r	0%	C	76

Int-C'2 10d

	15N %		Ratio
p	99%	A	13
q	70%	B	85
r	0%	C	2

GTX4 10d

	Incorporation %		Ratio
p	77%	A	49
q	38%	B	14
r	1%	C	37

11-hydroxy-Int-C'2 6d

	15N %		Ratio
p	81%	A	23
q	49%	B	31
r	2%	C	46

C2 6d

	15N %		Ratio
p	92%	A	1
q	56%	B	14
r	0%	C	85

11-hydroxy-Int-C'2 10d

	15N %		Ratio
p	87%	A	34
q	58%	B	40
r	3%	C	26

C2 10d

	15N %		Ratio
p	77%	A	49
q	42%	B	13
r	1%	C	38

C1 10d

	15N %		Ratio
p	90%	A	8
q	52%	B	13
r	0%	C	79

Table S-6. The detection limits (fmol/cell) for non-toxic species *A. insuetum* and *P. triestinum*

	A. insuetum *NIES-678*	*P. triestinum* *Ptri060930Ohi*
C1	< 0.74	< 0.04
C2	< 0.60	< 0.03
C3	< 6.19	< 0.30
C4	< 2.26	< 0.11
GTX1	< 1.43	< 0.07
GTX2	< 1.94	< 0.09
GTX3	< 2.52	< 0.12
GTX4	< 0.37	< 0.02
GTX5	< 1.02	< 0.05
GTX6	< 0.55	< 0.03
neoSTX	< 2.25	< 0.11
dcSTX	< 0.53	< 0.03
STX	< 0.24	< 0.01
Int-A'	< 0.05	< 0.002
Int-C'2	< 0.05	< 0.002
11-hydroxy-Int-C'2	< 8.55	< 0.41
Int-E'	< 4.59	< 0.22
Cyclic-C'	< 0.06	< 0.003

Optimization of Chromabond^R HILIC SPE conditions using the non-labelled standard

The SPE treatment for sample preparation prior to the HR-HILIC-quadrupole time-of-flight (Q-Tof) MS was modified from the previously described method developed for STXs [43]. Since the ZIC-HILIC^R SPE used in the original method is not commercially available so far, this reagent was replaced with Chromabond^R HILIC, a sorbent that has the same functional group. Moreover, the recovery rate of the biosynthetic intermediates was very low by the original method [43], which therefore was modified to permit the simultaneous analysis of both the labelled biosynthetic intermediates and the STXs. In the previous paper [15], cell pellets were lyophilized to recover the relatively less-polar compounds corresponding to the biosynthetic intermediates from early stages of the pathway, namely arginine, Int-A', and Int-C'2. For example, recovery of Int-C'2 was 5% by the original procedure [15]. Moreover lyophilization is time-consuming and difficult to apply for the multiple samples expected from a time-course study. Therefore, a clean-up procedure without lyophilization was developed. Simply performing the original procedure without lyophilization yielded low amounts of Int-A' and Int-C'2 (Supplementary Information Table S-2, Entry 1). The use of THF for application and washing of the sample yielded improved recovery of these biosynthetic intermediates. However, attempts at elution with 0.5 M acetic acid or 0.2 M formic acid directly after THF washing did not permit recovery of GTX4, C1, or C2 (Entry 2). Stepwise washing with THF, acetonitrile, and 95% acetonitrile containing 0.1% formic acid improved the elution of these STXs with 0.2 M formic acid (Entry 3). The recovery rates of the main toxins and the biosynthetic intermediates from 50 mg of Chromabond^R HILIC adsorbent were determined using a standard mixture prepared at a concentration range similar to that observed experimentally in the cell extracts of dinoflagellate cultures. The elution volume was set to 200 μL to enable direct analysis without the need for a concentration step, although the recovery rate could be improved by using a higher volume of elution solution.

Optimized sample clean-up for HR HILIC-ESI-Q-tof-MS and MS/MS

Aliquots of the harvested cultures were used to obtain cell counts by microscopy. The cultures (20 mL each) then were centrifuged at 1,700 *g* for 5 min at 4° C to pellet the cells. After removal of the supernatant, the pellet was transferred to a new micro-tube, re-suspended, and pelleted again by centrifugation. After removal of the supernatant, the pellet was re-suspended in 300 μL of 0.5 M acetic acid. Samples were stored at -30° C until use. After thawing on ice, the cell suspension was subjected to sonication (three cycles at 100 Hz, 40% amplitude, for 30 s on ice with 30 s intervals). The homogenate was centrifuged at 20,000 *g* for 5 min at 4° C. The supernatant of each sample was subjected to ultra-filtration (Ultra-Free C3LGC, 10,000-Da cut-off, Millipore) at 4° C. An aliquot (100 μL) of the resulting filtrate was transferred to a new tube and mixed with three volumes of THF. In parallel, a column of Chromabond^R HILIC adsorbent (50 mg, MACHEREY-NAGEL) was generated by packing into a disposable empty cartridge (syringe type cartridge (CS0111, S size) and frits (CF0003), Tomoe, Amagasaki, Japan) and conditioned with 200 μL of MilliQ water and 1 mL of THF. The sample was loaded onto the column and the column was sequentially washed with 500 μL of THF, 500 μL of CH_3CN, and 500 μL of CH_3CN/water/HCOOH (95:5:0.1, v/v/v). The column was eluted with 200 μL of 0.2 M HCOOH and an aliquot of the eluate (10 or 20 μL) was subjected to LC-MS.

For the MS/MS sample, a Chromabond^R HILIC polypropylene column (500-mg) was pre-conditioned with 1 mL of MilliQ water and 5 mL of THF. The total extract from a 20-mL culture was loaded onto the column, and the column then was sequentially washed with 3 mL of THF, 3 mL of CH_3CN, and 3 mL of CH_3CN/water/HCOOH (95:5:0.1, v/v/v). The column was eluted with 3 mL of 0.2 M HCOOH and the eluate was concentrated under a stream of nitrogen gas. After reconstitution with 100 μL of MilliQ water, the sample was filtered through a Cosmospin filter H (0.45 μm) and an aliquot of the eluate (10 μL) was subjected to LC-MS/MS .

The linearity, detection limit and retention time of standard of modified column-switching HR HILIC-ESI-Q-tof-MS

The standards (GTX1 and 4, GTX2 and 3, C1 and C2) were used as a mixture of the stereoisomers at a ratio of equilibrium. The stock solutions of STXs and the synthetic standards of the biosynthetic intermediates were stored in 0.05 M acetic acid and 0.5 M acetic acid, respectively, at −30 ° C. The stock solutions were diluted with 0.5 M acetic acid to yield dilutions appropriate to the sensitivity of each analysis. The linearity of the calibration curve was 0.997 (C2) – 0.999 (Int-C'2) in the range of 0. 4 – 4.3 μM (C2), 0.3 – 13.9 μM (GTX5), 0.5 – 10.4 μM (GTX4), 1.0 – 19.8 μM (C1), 0.1 – 1.0 μM (Int-A'), 0.05 – 2.5 μM (arginine), 0.01 – 1.0 μM (Int-C'2), and 0.25 – 1.0 μM (11-hydroxyl-Int-C'2, Int-E' and Cyclic-C'). The detection limit (S/N = 5) of the standard ranged from 0.01 μM (Int-C'2) to 0.3 μ M (C2) for 10- μ L injections. The retention times were 12.3, 13.2, 24.9, 25.1, 27.8, 30.1, 30.6, 31.0, 34.9, and 35.2 min for Int-C'2, Cyclic-C', Int-A', 11-hydroxyl-Int-C'2, arginine, C1, Int-E', GTX4, GTX5, and C2, respectively. The primary metabolites that might affect the mass spectra of these target molecules were analyzed to determine retention times. Notably, the primary metabolites acetyl-ornithine ($C_7H_{15}N_2O_3^+$, $[M+H]^+$ = *m/z* 175.1077) and citrullin ($C_6H_{14}N_3O_3^+$, $[M+H]^+$ =*m/z* 176.1030) either of which might affect the intensity of isotopomers for arginine ($C_6H_{15}N_4O_2^+$, $[M+H]^+$ =*m/z* 175.1190, m+1 $C_6H_{15}N_3{}^{15}N\ O_2^+$, $[M+H]^+$ =*m/z* 176.1160), eluted at 14.8 and 14.6 min, respectively. Thus, neither of these metabolites should influence the intensity of the isotopomers of arginine that eluted at 27.8 min. Acetyl-lysine ($C_8H_{17}N_2O_3^+$, $[M+H]^+$ =*m/z* 189.1234) eluted at 13.1 min, and therefore should not overlap with Int-A' (m+2, $C_8H_{19}N_2{}^{15}N_2O^+$, $[M+H]^+$ =*m/z* 189.1494), which itself eluted at 24.9 min.

Validation by the highly labelled sample mixed with the non-labelled standard

The two-month exposure was initiated in the same manner as the time-course study and passage was carried out three times for a total interval of two months (each passage was performed at 2 weeks). After 2 months, the cell cultures had achieved a cell density of 5 x 10^3 cells mL^{-1} and aliquots (60 mL each) were harvested by centrifugation at 890 *g* for 3 min at 4 °C. The supernatants were decanted and discarded. Each cell pellet was re-suspended in 300 μL of 0.5 M acetic acid and stored at −30 °C until analysis. The mono-isotopic ions of the non-labelled compounds were not detected except for arginine; the completely labelled isotopomers constituted the primary peak for each compound (Supplementary Information Fig. S-13). After ultrafiltration, the filtrate was mixed with a standard solution containing arginine, Int-A', Int-C'2, C1, C2, and GTX1-5 at final concentrations of 5.0, 0.5, 0.5, 4.9, 1.1, 7.6, 2.2, 0.8, 2.6, and 3.5 μM, respectively. The same procedure was performed for the control (without standard) sample and the standard mixture only. The recovery rates were calculated as follows: the area of mono-isotopic ion in the fortified sample minus that of the control was divided by that of the standard solution treated the same as the fortified sample. The samples for the validation study were prepared in triplicate. For the evaluation of matrix effects, the eluates of un-mixed cell extract from Chromabond^R HILIC sorbent were mixed with the standard solution. The matrix effects were calculated as follows: the area of mono-isotopic ion in the mixed sample minus the area of the control was divided by that of the standard solution. The values of recovery rate and matrix effect of Int-C'2 and those of arginine were used for Cyclic-C', 11-hydroxyl-Int-C'2 and Int-E'. The relative % was calculated as follows: the area of mono-isotopic ion in the mixed sample minus that of the control was divided by the sum of the areas of all isotopomers containing ^{15}N as observed in the EICs of the fortified samples.

Calculation of peak area removing the contribution of the naturally occurring stable isotopes

The concept of calculation

Arginine, Int-A', 11-hydroxy-Int-C'2, Int-E' and consist of C, H, O, and N. Int-C'2 consists of C, H, and N. STXs (GTX4, GTX5, C1, and C2) consist of C, H, O, N, and S. Therefore, the ^{15}N-labelled compounds contain not only the incorporated ^{15}N, but also the naturally occurring stable isotopes such as ^{13}C, ^{2}H, ^{17}O, ^{18}O, ^{15}N, ^{33}S, and ^{34}S. To obtain the newly synthesized isotopomer peak areas, it is necessary to remove the contribution by these naturally occurring stable isotopes. Since the natural abundances of the stable isotopes ^{13}C, ^{15}N, ^{18}O, and ^{34}S are 1.07, 0.364, 0.205 and 4.25%, respectively, two different isotopomers with the same nominal mass can exist for compounds containing these atoms. Since the mass spectrometer used in this study could not distinguish these isotopomers, the total of the theoretical natural abundances of two isotopomers having the same nominal mass with different formulae was used. For example, the natural abundances for C2 $[M–SO_3]^+$ were m/z 396.0932 (100.0%), 397.0966 (10.8%), 398.0890 (4.5%), 397.0902 (2.6%), and 398.0975 (1.6%). The theoretical natural abundance for m+1 (m/z 397) was 13.4% and that for m+2 (m/z 398) was 6.1%. Please see Table S-3 for the example of the calculation.

Quantitation of the biosynthetic intermediates, the shunt product, and STXs in non-toxic and toxic dinoflagellates using column-switching HILIC- MS/MS (MRM)

Aliquots of cultured cells (15 mL of *A. insuetum* (NIES-678) at 5.7 × 10^3 cells mL^{-1}, 20 mL of *P. triestinum* (Ptri060930Ohi) at 1.2 × 10^5 cells mL^{-1}, and 10 mL of *G. catenatum* (GC-18) at 2.8 × 10^3 cells mL^{-1}) were centrifuged at 1700 *g* (*A. insuetum* and *P. triestinum*) or 2300 *g* (*G. catenatum*) for 5 min to pellet the cells. The pellets were re-suspended in 300 μL of 0.5 M acetic acid, and the cells were disrupted by sonication (3 times at 100 Hz for 30 sec on, 30 sec off) on ice. The suspensions then were centrifuged (20,000 *g* for 5 min at 4 ºC), and the resulting supernatants were subjected to ultra-filtration (Ultra-Free C3LGC, 10-kDa cut-off, Millipore). Triplicate samples were prepared for each strain. An aliquot of each filtrate was subjected to quantitative analysis by HILIC-MS-MRM (10 μL) using an API2000 triple-quadrupole tandem MS equipped with an ESI source, as described previously [20].
